# Steered molecular dynamics simulations reveal critical residues for (un)binding of substrates, inhibitors and a product to the malarial M1 aminopeptidase

**DOI:** 10.1371/journal.pcbi.1006525

**Published:** 2018-10-31

**Authors:** Daniel S. Moore, Conor Brines, Heather Jewhurst, John P. Dalton, Irina G. Tikhonova

**Affiliations:** 1 School of Pharmacy, Medical Biology Centre, Queen’s University Belfast, Belfast, Northern Ireland, United Kingdom; 2 School of Biological Sciences, Medical Biology Centre, Queen's University Belfast, Belfast, Northern Ireland, United Kingdom; Max Planck Institute for Biophysical Chemistry, GERMANY

## Abstract

Malaria is a life-threatening disease spread by mosquitoes. *Plasmodium falciparum* M1 alanyl aminopeptidase (*Pf*M1-AAP) is a promising target for the treatment of malaria. The recently solved crystal structures of *Pf*M1-AAP revealed that the buried active site can be accessed through two channel openings: a short N-terminal channel with the length of 8 Å and a long C-terminal channel with the length of 30 Å. It is unclear, however, how substrates and inhibitors migrate to the active site and a product of cleavage leaves. Here, we study the molecular mechanism of substrate and inhibitor migration to the active site and the product release using steered molecular dynamics simulations. We identified a stepwise passage of substrates and inhibitors in the C-terminal channel of *Pf*M1-AAP, involving (I) ligand recognition at the opening of the channel, (II) ionic translation to the ‘water reservoir’, (III) ligand reorientation in the ‘water reservoir’ and (IV) passage in a suitable conformation into the active site. Endorsed by enzymatic analysis of functional recombinant *Pf*M1-AAP and mutagenesis studies, our novel ligand-residue binding network analysis has identified the functional residues controlling ligand migration within the C-terminal channel of *Pf*M1-AAP. Furthermore, from unbinding simulations of the Arg product we propose a charge repulsion as the driving force to expel the product out from the N-terminal channel of *Pf*M1-AAP. Our work paves the way towards the design of a novel class of P*f*M1-AAP inhibitors based on preventing substrate entry to the active site.

## Author summary

Malaria is a tropical disease caused by infections with protozoan parasites of the genius *Plasmodium*. Currently, the disease results in over 400,000 human deaths per year worldwide and future prevention and treatment is at risk due to the rise of drug-resistant parasites. Plasmodium falciparum M1 alanyl aminopeptidase is an enzyme involved in the terminal stages of haemoglobin digestion by the intra-erythrocytic stages of the parasite. Chemical inhibition of the enzyme activity prevents the supply of amino acids for parasite development within the blood cells and causes death of malaria parasites. Despite being a promising antimalarial drug target, this enzyme was challenging for inhibitor search using random screenings. One of the reasons is the poorly accessible active site. This project involves computer simulations of the enzyme crystal structures to understand ligand recognition and binding to the buried active site. We demonstrate that electrostatic interactions regulate ligand migration to the active site and offer opportunities for rational design of novel inhibitors.

## Introduction

Malaria is a life-threatening tropical disease caused by parasites of the genus *Plasmodium* and spread by *Anopheline* mosquitoes. Malaria remains one of the top pernicious infectious diseases of humans with 212 million infections and 429,000 deaths each year, predominately in children under 5 years of age in sub-Saharan Africa [1]. The prevention and treatment of malaria is under threat due to the spread of parasites resistant to the current frontline antimalarial drugs [2,3]. As a result, there is a pressing need for new antimalarial therapies.

*Plasmodium falciparum* is the species of the malaria parasite that causes the majority of human disease and the highest mortality [4]. For the development of the intra-erythrocyte stages of parasite, the degradation of host cell hemoglobin is necessary to support protein synthesis and metabolism [5]. Hemoglobin is initially degraded to di- and tri-peptides by numerous parasite proteases within a specialized food or digestive vacuole [6]. Peptides generated by this process are then transported into the parasitic cytosol, where hydrolysis to free amino acids takes place with a help of the cytosolic M1 alanyl aminopeptidase (*Pf*M1-AAP) as well as several other exo-metallo-aminopeptidases [5,7]. *Pf*M1-AAP has a particularly broad substrate specificity cleaving peptide bonds involving hydrophobic, basic and aromatic amino acids [8–10]. Blocking of *Pf*M1-AAP activity with inhibitor compounds such as Bestatin prevents the supply of amino acids for parasite development, therefore, and kill parasites both *in vitro* and *in vivo* making this an attractive strategy for design of novel anti-malarial therapies [11].

**Fig 1:**
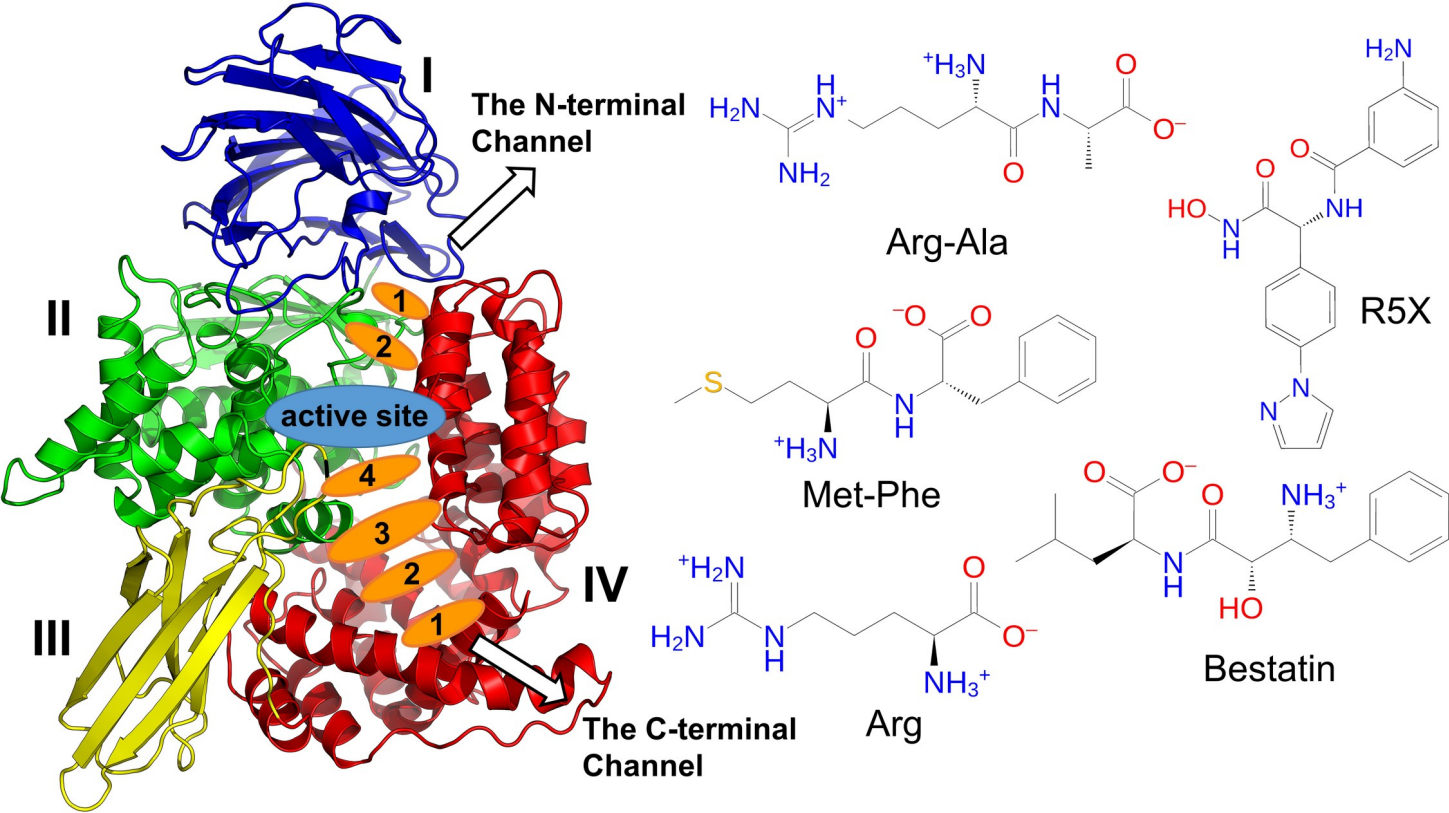
The *Plasmodium falciparum* M1 alanyl aminopeptidase (*PfM1*-AAP) with the substrates (Arg-Ala and Met-Phe), inhibitors (Bestatin and R5X) and Arg product. The *Pf*M1-AAP domains are coloured and labelled. The long C-terminal and short N-terminal channels and the position of residues selected for calculation of R_gyr_ are shown.

Information of the enzyme three-dimensional structure is crucial for an understanding of the molecular basis of substrate recognition and compound inhibition and essential for structure-based inhibitor design. In the past decade, 25 crystal structures of *Pf*M1-AAP in the ligand-bound and unbound forms were solved, revealing a common N-fold architecture with bacterial aminopeptidases and binding interactions with inhibitors and products. Structurally, *Pf*M1-AAP includes 26 α-helices and 7 β-sheets that form four domains (Fig 1). The active site with the catalytic Zn^2+^ ion coordinated by His496, His500, Glu519 and a catalytic water molecule is buried in the core of the protein. The crystal structure complexed with the inhibitor Bestatin [9], a natural analogue of dipeptide Phe-Leu, reveals 8 hydrogen bonds with the active site, where the Phe moiety is in the P1 hydrophobic pocket composed of Val459, Met462, Tyr575 and Met1034, and the Leu moiety sits in the P1’ pocket of Val493, Val523 and Thr492. From the Bestatin-*Pf*M1-AAP complex substrate binding was readily predicted by molecular docking programs.

The crystal structure analysis shows that the active site is buried within the enzyme and can be accessed through two channel openings: a short N-terminal channel with the length of 8 Å and located at the interface between domains I and IV, and a long C-terminal channel with the length of 30 Å within domain IV [9] (Fig 1). Because the crystal structures show a static view of *Pf*M1-AAP-ligand bound state, it remains unclear as to how substrates and inhibitors migrate to the buried active site and how products leave. Moreover, it is unknown whether the migration of substrates is via simple diffusion or regulated by *Pf*M1-AAP and which channel allows the substrates to occupy a suitable configuration to fit into the active site for the catalytic reaction. Understanding the mechanisms of substrate entry and exit would set the groundwork for the development of a novel class of *Pf*M1-AAP inhibitors aimed at blocking these events.

In the present study, we have selected two ligands as substrates, Met-Phe and Arg-Ala, two inhibitors, Bestatin and R5X (Fig 1) and the Arg product to examine substrate and product migration to and from the active site along both channels using multiple steered molecular dynamic (sMD) simulations [12]. Initially, the migration of the ligands in each channels was explored in short 30 ns multiple sMD simulations and then extended to a long 100 ns sMD simulation at a reduced speed. A table with the simulation systems and details of the sMD simulations is shown in the Supporting Information (S1 Table). Such an approach allowed enhanced sampling of ligand configurations along the binding pathways and highlighted ligand-protein interactions involved in the migration. We have also performed classical MD (cMD) simulations of P*f*M1-AAP and exploited the availability of >20 crystal structures to further extend the results of the sMD simulations. Our developed ligand-residue binding network together with other MD trajectory analyses highlight a stepwise passage of substrates and inhibitors from the external aqueous environment into the P*f*M1-AAP active site via the C-terminal channel, the mechanism of the product release via the N-terminal channel and the role of water molecules. Our computer simulations of the ligand migration were endorsed by enzymatic assays using functionally-active wild-type recombinant P*f*M1-AAP and a specific variant, P*f*M1-AAP Arg969Ala, and thus provide the groundwork for the design of new P*f*M1-AAP inhibitors for the treatment of malaria that prevent substrate entry and/or product exit from the active site.

## Results

### Migration of substrates, inhibitors and a product from the *Pf*M1-AAP active site

The long C-terminal and short N-terminal channels are the main routes for ligands to reach the deeply buried active site of *Pf*M1-AAP. To explore the dynamic properties of the two channels, we first performed cMD simulations of the ligand-unbound *Pf*M1-AAP form and calculation of the radius of gyration (R_gyr_) at several areas of the channels. In particular, the average R_gyr_ was calculated in four regions of the long C-terminal channel and in two regions of the short N-terminal channel. The position of residues selected for calculation of R_gyr_ is shown in Fig 1 and the residue number of Cα atoms is defined in the Methods section. The external opening and the start of the C-terminal channel cavity have the R_gyr_ of 8.9 and 8.6 Å, respectively. The channel then enlarges reaching the R_gyr_ of 12,7 Å and reduces again up to the R_gyr_ of 9.7 Å in the cMD simulations (Table 1). In the case of the N-terminal channel, the R_gyr_ is 6 and 7 Å at the internal and external openings, correspondently. Thus, the C-terminal channel is wider at all the length of the channel compared to the N-terminal channel. Fluctuations in the R_gyr_ are minor for both the channels, indicating that the channels are relatively rigid in the cMD simulations (Table 1).

**Table 1:** Fluctuation of the radius of gyration (R_gyr_) at several locations of the C- and N-terminal channels (see Fig 1) in the cMD simulations of the empty *Pf*M1-AAP and during the ligand passage in the 100ns sMD simulations.

*Pf*M1-AAP Systems	C-terminal Channel								N-terminal Channel			
	Opening		Around Arg969		Around Water Reservoir		Around Arg489		Internal Opening		External Opening	
	R_gyr_, Å	Δ, Å	R_gyr_, Å	Δ, Å	R_gyr,_ Å	Δ, Å	R_gyr,_ Å	Δ, Å	R_gyr,_ Å	Δ, Å	R_gyr,_ Å	Δ, Å
Empty	8.9±0.2		8.6±0.1		12.7±0.2		9.7±0.1		6.0±0.1		6.9±0.2	
Arg-Ala	9.2±0.1	0.3	9.1±0.1	0.5	13.4±0.1	0.7	10.5±0.1	0.8	8.1±0.1	2.0	8.9±0.2	2.0
Met-Phe	9.6±0.1	0.7	8.9±0.2	0.3	13.0±0.1	0.3	10.3±0.1	0.6	7.9±0.2	2.0	8.6±0.2	1.7
Bestatin	8.5±0.1	0.4	8.7±0.1	0.1	13.1±0.1	0.4	10.3±0.1	0.6	7.8±0.1	1.8	8.3±0.2	1.5
R5X	9.1±0.1	0.2	9.1±0.3	0.5	13.0±0.3	0.3	10.3±0.1	0.6	8.2±0.1	2.2	8.1±0.2	1.2
Arg	8.5±0.1	0.4	8.6±0.1	0.1	13.1±0.1	0.4	10.4±0.1	0.7	7.8±0.2	1.0	7.9±0.2	1.0

We then compared the variation in the R_gyr_ values during the passage of the ligand in the sMD with the cMD simulations. Table 1 shows the results from the 100 ns sMD simulations and S2 Table in the Supporting Information from the 30 ns sMD simulations. The change in the R_gyr_ values for the C-terminal channel is smaller (±0.1–0.8 Å) compared to the N-terminal channel (±1.2–2.0 Å), indicating that small conformational changes are needed in the C-terminal channel to pass the ligands. By contrast, the N-terminal channel requires more structural changes, which increase with the size of the ligand (Arg versus Arg-Ala, Table 1). Furthermore, we have noted that the substrates typically adopt a bent conformation, resulting in the interaction between the C- and N-terminal ends, as they migrate through the N-terminal channel. This bent conformation has a strain energy of 5 kcal/mol compared to the extended conformation of the substrates observed through the passage of the C-terminal channel. Pulling of the Arg product requires the smallest change in the R_gyr_ of the N-terminal opening. The R_gyr_ data demonstrates that the migration of the substrates and inhibitors through the N-terminal channel is more hindered compared to the migration of the smaller Arg product.

**Fig 2:**
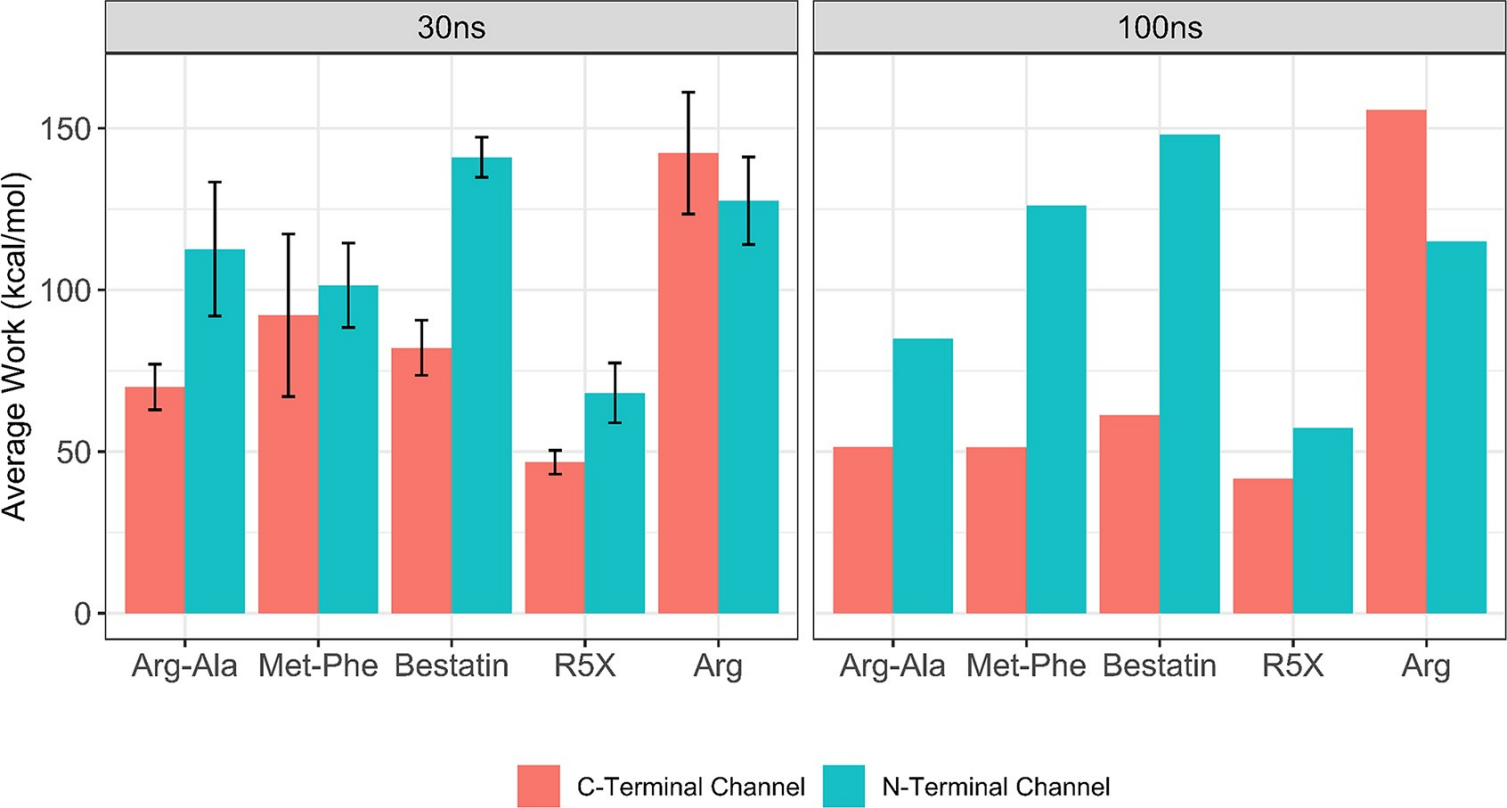
Work values from the six replicate 30 ns and single 100 ns sMD simulations indicate the preference of the substrate and inhibitors to migrate through the C-terminal channel and for the product though the N-terminal channel.

To further gain insight into the preferable channel for ligand migration, we calculated the work that ligands undertake while passing through the N- and C-terminal channels using the 30ns and 100ns sMD trajectories (Fig 2). From the multiple 30ns simulations the results show that Arg-Ala, Bestatin and R5X require more work, 113±21, 141±6 and 68±9 kcal/mol to pass through the N-terminal channel compared to the C-terminal channel, 70±7, 82±9 and 47±4 kcal/mol. Less difference in the average work is observed for Met-Phe, 101±13 and 92±25 kcal/mol to unbind through the N- and C-terminal channels, respectively. The work for Arg unbinding is 142±19 kcal/mol in the C-terminal channel and 128±14 kcal/mol in the N-terminal channel, indicating the greater ease to pull Arg through the N-terminal channel. From the single 100 ns runs (Fig 2), the work required for the migration through the N- and C-terminal channels for the large and small ligands is even more distinct, suggesting that in longer simulations the observed pattern of work difference is more notable.

Although, more sMD simulation replicates are required to provide accurate calculation of the work, our data notably indicates that the migration of the substrates and inhibitors through the C-terminal channel is more favourable than through the N-terminal channel. In contrast, our simulations demonstrate that the product leaves the active site from the N-terminal channel. This scenario is in agreement with the proposed mechanism of substrate binding derived from the P*f*M1-AAP crystal structure analysis [9].

### Migration to the active site involves specific interactions with residues of the C-terminal channel

**Fig 3:**
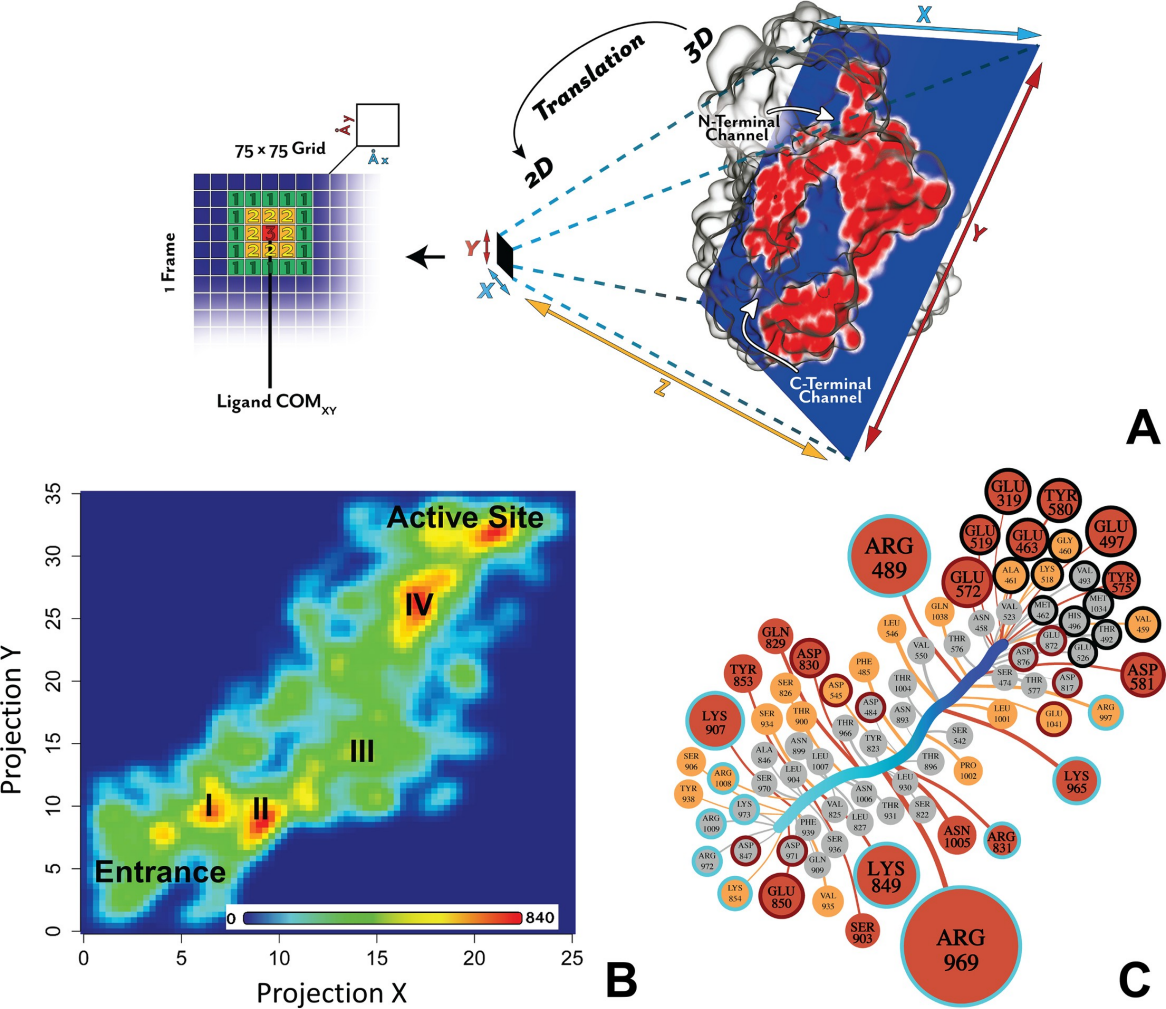
Ligand migration through the C-terminal channel. **A:** Definition of 2D projection to create a ligand occupancy map. The plane defined by the cross section through the centre of the C-terminal channel was used to map the 3D coordinates of the centre of mass (COM) of all the ligands into the X and Y projections. The plane is in blue and the protein slice in this plane is in red. The ligand occupancy map was calculated using a 75x75 grid matrix. For each frame of each simulation, a 2D coordinates of the ligand COM is counted and located into one of the grid cell. **B**: The 2D ligand occupancy map from the all 30-ns sMD trajectories for each ligand. The entrance of the C-terminal channel and the active site are labelled. The transient stable states corresponding to interactions with Glu850 and Lys907 (I); Lys849 and Asp830 (II); Arg969 and water reservoir (III) and Arg489 and Lys965 (IV). Red indicates high occupancy and blue low occupancy. Colouring is linear and values in the bar in counts. **C**: The ligand-residue binding network (LRBN) derived from the ligand-residue interaction energy of the all sMD trajectories. Nodes are residues interacting with the ligand during the migration. The size and colour of the nodes correspond to the strength of interaction. The 25% and 50% strongest interactions and the remaining frequently occurring interactions are in red, yellow and grey, respectively. Edges reflect the period of occurrence of a ligand-residue interaction; thicker edges show prolong interactions. The average ligand path is shown as a thick light-to-dark-blue-changing colour line. The active site, positively-charged and negatively-charged residues are circled in black, blue and red, respectively.

We next focused on the binding process of substrates and inhibitors as they migrate in the C-terminal channel to the active site. To examine a molecular mechanism of ligand migration through the channel entrances, we created the ligand occupancy map from the sMD trajectories. The ligand occupancy map represents a 2D population histogram, defined by the cross section through the channel centre (Fig 3A), which shows the relative position of the ligands in the channel (Fig 3B) during a simulation run. Using this approach, we have identified three main high-occupancy areas (the most frequently stayed regions) within the C-terminal channel, two areas at the entrance of the channel and one area in close proximity to the active site (coloured red). At the centre of the channel, the occupancy area is wider and less dense (coloured green). This reveals that the ligands sample a larger space in the wide part of the C-terminal channel (R_gyr_ = 12,7 Å). Overall, the ligand occupancy map indicates that the migration of the substrates and inhibitors along the C-terminal channel involves several transient states that are stabilized by interactions with specific residues of the channel.

To identify the amino acid residues that form interactions with the ligand and their importance in ligand migration we developed a ligand-residue binding network (LRBN) based on the calculation of the interaction energy between the ligand and a residue of the binding channel. LRBN aggregates information about ligand-residue interactions from the sMD simulated replicates of all ligand-protein systems in the form of weighted nodes and edges. The LRBN diagram of the migration through the C-terminal channel is presented in Fig 3C. A node represents a residue forming the interaction with the ligand with the node size corresponding to the strength of the average ligand-residue interaction energy calculated from the sMD simulations. The node colour ranks the residues into three groups based on the strength of the interaction energy; thus, red, yellow and grey nodes are the top 25% and 50% of strong ligand-residue interactions and the remaining frequently-occurring interactions, respectively. The nodes are connected to the average ligand binding pathway through the edge at the point of the highest interaction energy. The edge width is proportional to the timeframe of the ligand-residue interaction occurrence and, thus, the thick edge shows a prolonged interaction. Overall, this approach effectively creates a graph of a generic ligand passage through the channel to the active site highlighting residues that form strong and prolonged interactions with a ligand.

From LRBN (Fig 3C), it is evident that the ligand migration through the C-terminal channel is orchestrated by several charged residues, represented by the large nodes. In particular, five positively charged residues, Arg969, Arg489, Lys849, Lys907 and Lys849, and four negatively charged residues, Glu850, Asp830, Glu572 and Asp581 play an important role in the ligand migration and stabilization of the ligand transient stable states shown in the ligand occupancy map (Fig 3A). The specific role of these residues is explained in the following sections.

#### Ligand recognition by charged residues of the C-terminal channel opening

**Fig 4:**
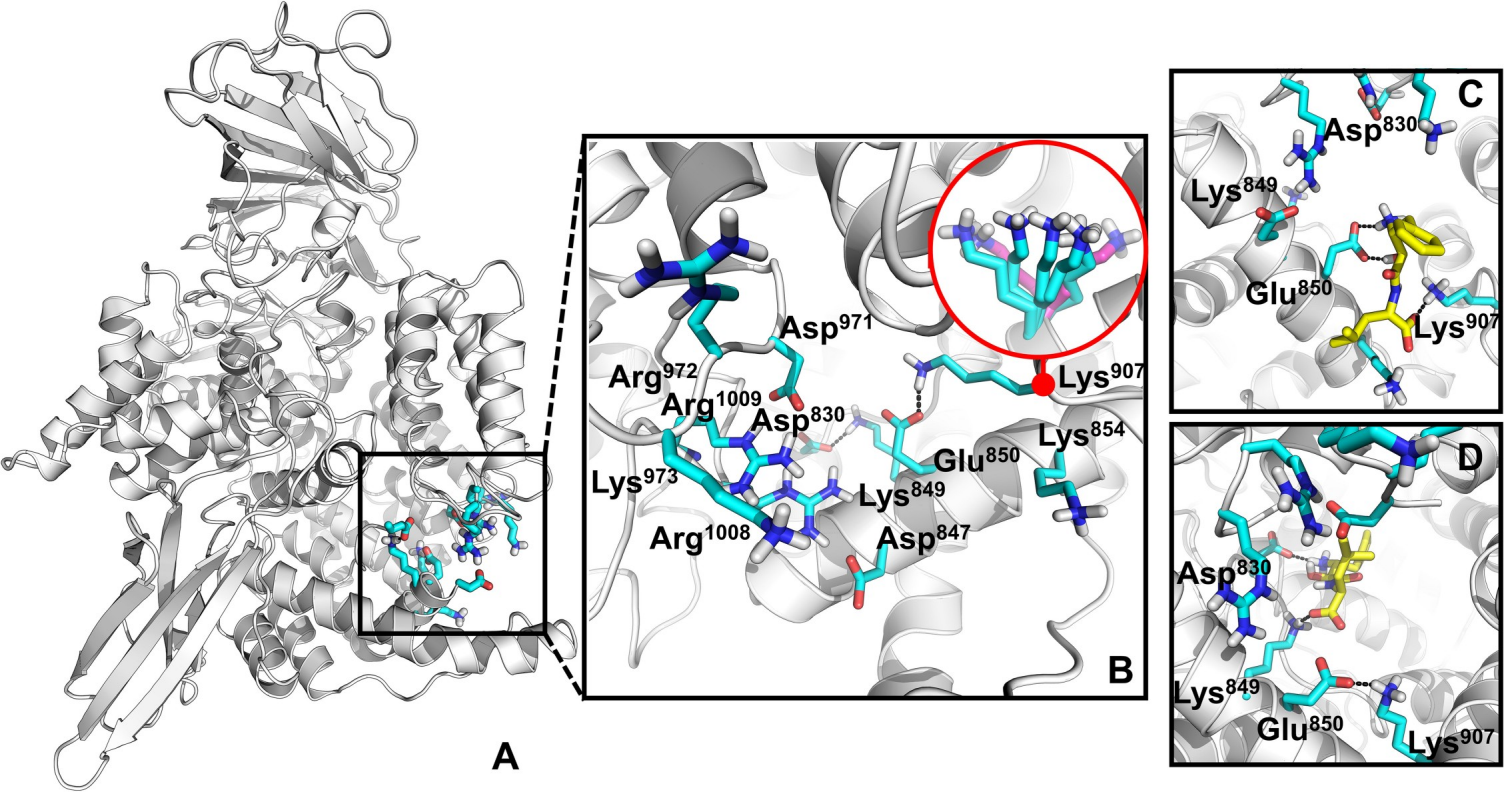
The C-terminal channel opening is made up of positively and negatively charged residues. **A**: The overall structure of P*f*M1-AAP with the visualized charged residues of the channel entrance that coordinate the initial migration of the substrates and inhibitors. **B:** The zoomed view of the channel entrance. The flexibility of the Lys907 side chain is shown in the circle; conformations captured in sMD are in cyan and conformations observed in the crystal structures are in purple. **C:** Transient stable binding of Bestatin with Glu850 and Lys907. **D:** Transient stable binding of Bestatin with Lys849 and Asp830. The salt bridges are shown in a black-dotted line.

The opening of the long C-terminal channel is lined by several positively and negatively charged residues as shown in Fig 4A and 4B. From our sMD simulations, these residues initially attract the carboxyl and ammonium groups of the ligands through electrostatic interactions. With the inhibitor R5X, which lacks these groups, the interactions form via the hydroxamic acid and aromatic amino group, respectively. Thus, typically, ligands form initial brief contacts with Arg1008, Arg1009, Lys973, Asp971 and Asp847 before the ammonium group of the ligands engages in a first strong interaction with Glu850 as identified by the LRBN diagram (Fig 3C).

In the simulations Glu850 forms a salt bridge with Lys907, which breaks and reforms intermittently (Video 1S). Glu850 bound to the ammonium group of the ligand invokes the passage of the ligand to Lys907 and the formation of interactions between the carboxyl group of the ligand and Lys907. The side chain of Lys907 with the ligand then rotates from the opening into the cavity of the channel, moving the ligand into the channel (Fig 4C). The dynamic behaviour of the Lys907 side chain is evident from the available crystal structures of P*f*M1-AAP (Fig 4B). The LRBN diagram highlights strong interactions between Lys907 and the ligands. From our sMD simulations it appears that Glu850 serves as a ligand recognition point, while Lys907 plays a leading role in facilitating ligand movement from the aqueous external environment (parasitic cytosol) into the long C-terminal channel.

Within the channel cavity, Lys907 passes the substrate to Lys849 and Asp830 (Video 1S). These residues form prolonged interactions with the carboxyl and ammonium groups of the ligands, respectively (Fig 4B and 4D). At this point, the ligand is fully immersed into the channel cavity. Unlike Lys907, the Lys849 and Asp830 residues do not move the ligand farther away but instead, hold the ligand in place for the next interaction with Arg969 (see the next section).

Therefore, our sMD trajectory analysis indicates that the first two high-occupancy areas of the ligand (Fig 3B) are due to the interactions with Glu850 and Lys907 (I); and Lys849 and Asp830 (II). These residues regulate recognition and initial passage/migration of the ligands into the channel cavity.

#### Arg969 acts as a key ‘ionic regulator’ within the C-terminal channel

**Fig 5:**
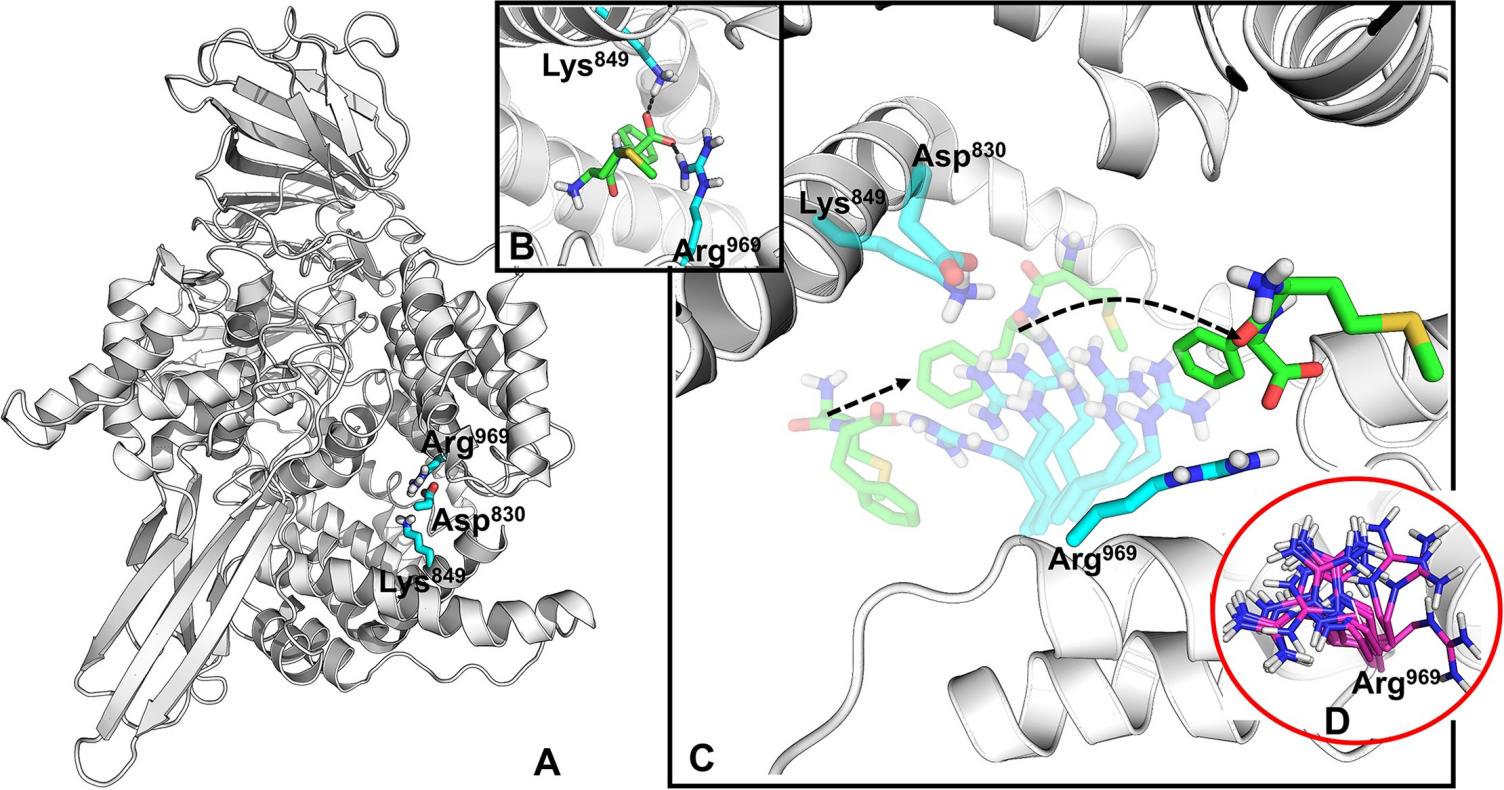
The regulatory Arg969. **A**: The overall structure of P*f*M1-AAP with the visualized Asp830, Lys849 and Arg969. **B**: Lys849 passes the Met-Phe substrate to Arg969. **C**: The migration of the Met-Phe substrate to the deeper cavity of the C-terminal channel with the help of Arg969. **D**: The various conformations of Arg969 from the P*f*M1-AAP crystal structures.

The next progression of ligands from Lys849 and Asp830 is controlled by Arg969 (Fig 5A and 5B). In both cMD and sMD simulations Arg969 moves freely, swinging from the channel entrance- to the active site-facing orientations for the majority of time. In the sMD simulations, while in the channel entrance orientation Arg969 chelates the carboxyl group of the ligand through a salt bridge, replacing the interactions with Lys849 and moves to its active site orientation, effectively pulling the ligand deeper into the channel cavity (Fig 5C and Video 2S). The distance travelled by the ligand during the swinging of Arg969 is around 8 Å. Indeed, in the ligand occupancy map the Arg969 interaction falls within the wide transient state III (Fig 3B) and LRBN shows the strongest and prolonged interaction is with Arg969 (Fig 3B), demonstrating its dominant role in regulating ligand migration.

To further clarify the importance of Arg969 and its movement during the ligand migration we have conducted cMD simulations of the biosystem with the starting point of the Arg969 side chain in the channel entrance orientation, where it forms a first contact with the ligand as shown in Fig 5C. Indeed, during the simulation the Arg969 side chain was found to move towards the active site, dragging the ligand into the channel. The result of the unbiased simulation further supports that Arg969 actively pulls ligands deeper into the channel cavity. The different conformations of the Arg969 side chain pointed to the active site (in 16 crystal structures) and the channel entrance (in 9 crystal structures) were found in the crystal structures, Fig 5D, revealing the high mobility of Arg969.

From the simulations we propose that Arg969 acts as an ‘ionic regulator’ that interacts with a charged group of the substrate or inhibitor and gears the ligand to the central enlarged cavity of the C-terminal channel.

#### Substrates find a suitable orientation in a water reservoir before entering the active site

**Fig 6:**
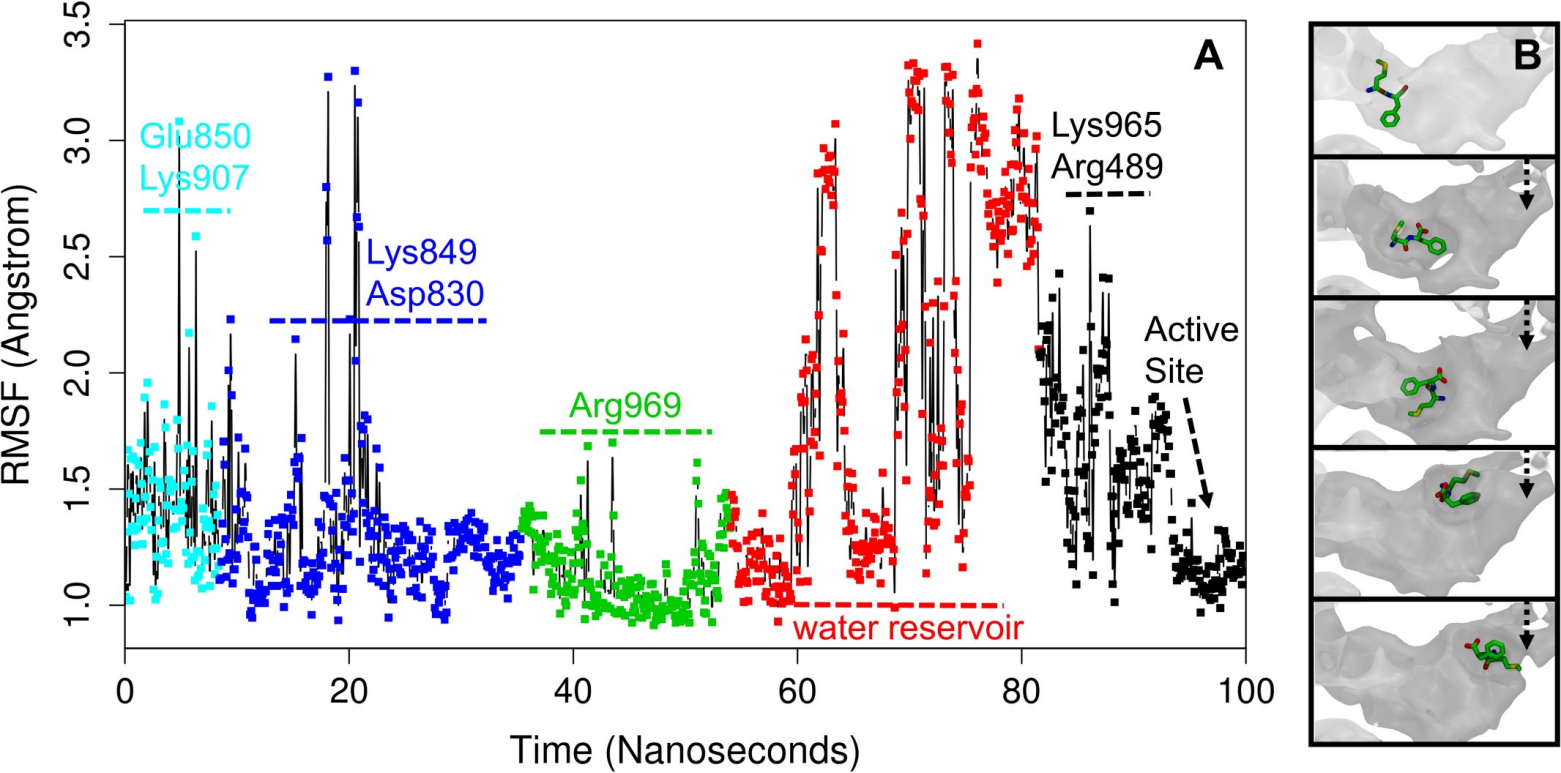
Ligand migration in the water reservoir. **A:** Root-mean-square fluctuation (RMSF) of the ligand (the Met-Phe substrate as an example) along the sMD trajectories. **B:** The various orientation of the Met-Phe substrate in the water reservoir. The part of the C-terminal channel involving the water reservoir is shown in the surface-like representation.

Arg969 brings the ligands to the centre of the enlarged water-filled cavity, which we term ‘the water reservoir’. Initially, the ligands are released from Arg969 and become fully solvated, losing most hydrogen bonds with the enzyme, which permits the ligands to become significantly more mobile, as shown by the root-mean-square fluctuation (RMSF) plot of the Met-Phe substrate as an example (Fig 6A). The high mobility of the ligand in the water reservoir also corresponds to transient state III in the ligand occupancy map (Fig 3B) and the absence of any strong and prolonged interactions with the enzyme in this part of the binding pathway is demonstrated by small contribution of residues in the LRBN diagram (Fig 3C). Free re-orientation, turning and flipping, allows the ligand to find a suitable orientation and conformation that would complement to the active site (Fig 6B and Video 3S). Finding a favourable orientation and conformation for the ligands may take several attempts. In this scenario, the water reservoir effectively acts as a chamber that holds the ligand, while it attempts to bind to the active site.

We previously proposed that the water reservoir provides water molecules for the efficient catalysis and hydration of the peptide bond [9] because a water molecule coordinates the Zn^+2^ cation together with His496, His500 and Glu519, and acts as the nucleophile to attack the carbonyl carbon of the substrate [9,13]. The water reservoir was also suggested to act as a ‘waiting-room’ or ‘vestibule’ [13], where peptides reside awaiting their entry into the active site for cleavage. Here, based on the results of our sMD simulations we suggest a new additional function for the water reservoir, where a ligand finds a suitable configuration to fit the active site. Moreover, from the above observations and size of the water reservoir (R_gyr_ = 13 Å) we can make assumptions on the largest possible peptides that could migrate up the channel–dipeptides/tripeptides.

**Fig 7:**
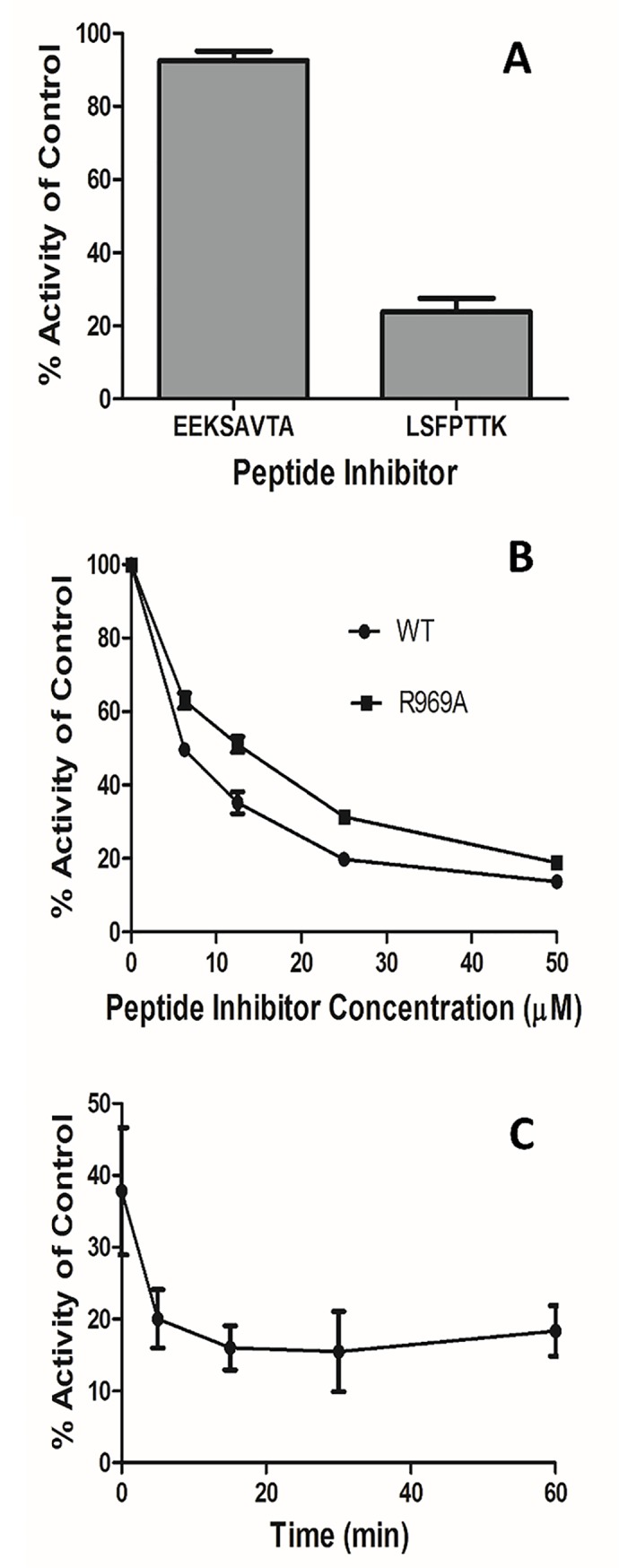
Enzymatic analysis of *Pf*M1-AAP and the variant PfM1-AAP Arg969Ala. **A**. Peptides EEKSAVTA and LSFPTTK (50 μM) were incubated with functionally-active recombinant *Pf*M1-AAP for 10 mins before the addition of the fluorogenic aminopeptidase substrate L-Arg-NHMec (5 μM). The release of the fluorescent moiety (-NHMec) was monitored over time in a fluorimetre (excitation 370nm, emission 460 nm). Relative fluorescent units within the linear phase of the reactions were calculated and no inhibitor controls taken as 100%. LSFPTTK inhibits the cleavage of the fluorogenic substrate, while the E-peptide does not. **B**. Concentration dependent competitive inhibition of *Pf*M1-AAP and the variant *Pf*M1-AAP Arg969Ala is observed the peptide LSFPTTK but this is less effective for the variant enzyme. **C**. Competitive binding assays performed by adding the fluorogenic substrate L-Arg-NHMec at various time-points (5, 10, 30 and 60 mins) after the addition of the peptide LSFPTTK (50 μM) to *Pf*M1-AAP reveals a constant level of inhibition suggesting that the L-peptide can enter the C-terminal channel but is not cleaved by the enzyme.

To validate the above proposed mechanism of ligand binding and migration (steps 1–3), we used a functionally-active recombinant *Pf*M1-AAP [8,9] and a fluorogenic aminopeptidase substrate (L-Arg-NHMec) to conduct competitive binding assays for two haemoglobin-derived peptides (L-SFPTTK and E-EKSAVTA) [14]. We found that the L-peptide competitively inhibits the cleavage of the fluorogenic substrate, while the E-peptide does not (Fig 7A). This is in agreement with earlier studies showing that *Pf*M1-AAP does not cleave substrates with N-terminal Glu or Asp [8,9[. Our computer simulations show that the first strong interactions formed by the ligand in *Pf*M1-AAP are with Glu850, the recognition site. We propose that the negatively charged N-terminal residue of the E-peptide repulses with Glu850, preventing the entrance to the channel.

Given that Arg969 of the C-terminal channel forms the strongest contacts with the ligands in our computer simulations (Fig 7B), we prepared a variant recombinant form of *Pf*M1-AAP with Arg969 replaced by Ala. While the rate of cleavage of the substrate H-Arg-NHMec was unaffected by the Arg969Ala substitution, competitive binding experiments with the *Pf*M1-AAP Arg969Ala variant showed that the inhibition of the fluorogenic substrate by the L-peptide is less effective compared to the wild type, *Pf*M1-AAP. From this experiment, it is evident that the L-peptide binds to the C-terminal channel and blocks the access to the channel for the fluorogenic substrate.

Interestingly, competitive binding assays performed by adding the substrate L-Arg-NHMec at various time-points after the L-peptide was mixed with the wild-type enzyme shows that the L-peptide binds to *Pf*M1-AAP but it is not cleaved (Fig 7C). This data suggests that the L-peptide can enter the C-terminal channel but does not reach the active site or is not in the suitable conformation to fit to the cleavage site. From our simulations, it becomes clear that such large peptide of the 14 Å length does not have enough space to re-orientate in the water reservoir of the 8 Å length with R_gyr_ of 13 Å to occupy a suitable conformation for the entrance to the active site.

#### Entry to the active site

**Fig 8:**
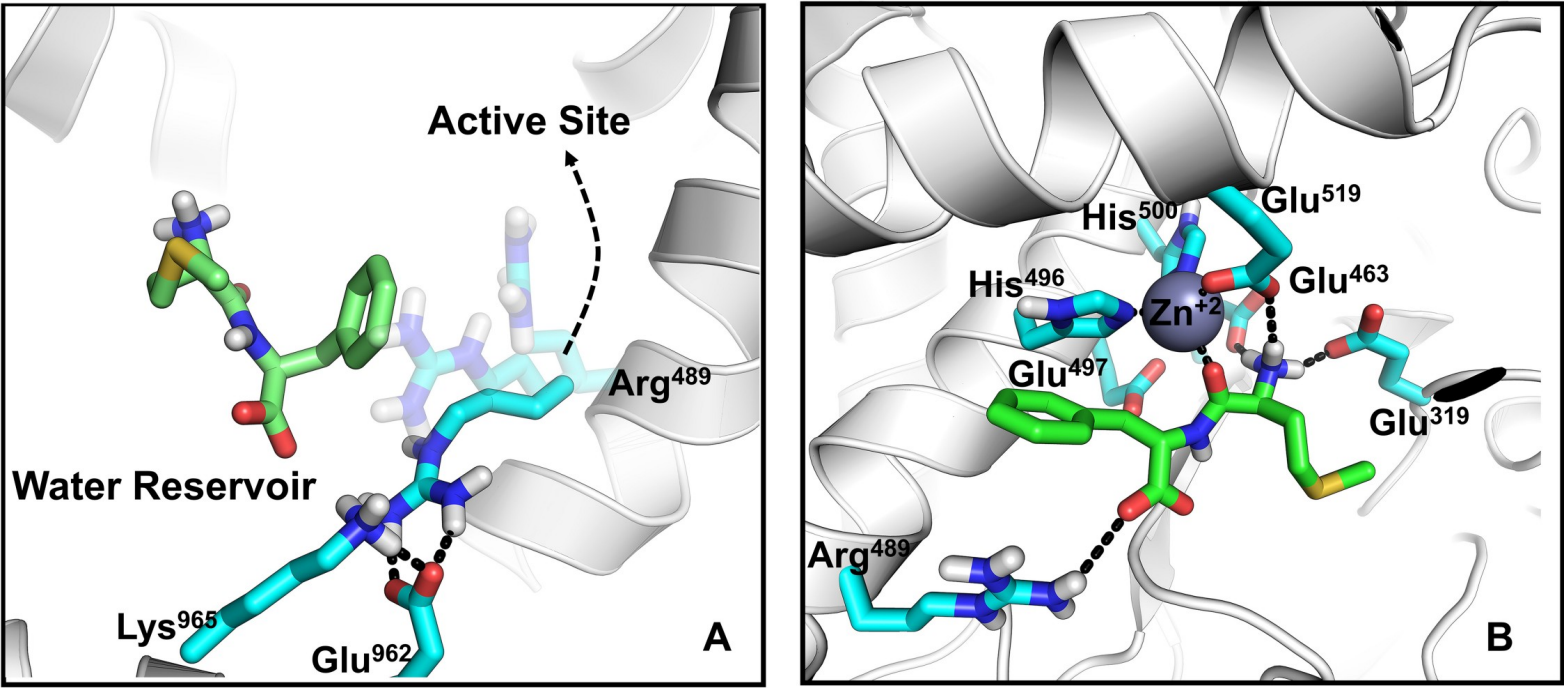
Migration of the ligand from the water reservoir to the active site of P*f*M1-AAP. **A:** Met-Phe approaches the positively charged Lys965-Glu962-Arg489 cluster to form a salt bridge with Arg489 that brings Met-Phe to the active site. Rotation of Arg489 to the active site is shown with two transparent Arg489 side chains. **B:** The final position of Met-Phe in the active site. The salt bridges and hydrogen bonds are shown in a black-dotted line.

The next progression of the ligand involves interactions with Arg489 and Lys965. Periodically, in the absence of the ligand, Arg489 and Lys965 each form a salt bridge with Glu962. With Arg489 orientated away from the active site, the Lys965 and Glu962 maintain a balanced neutral electrostatic surface potential that has a low propensity to attract ligands (Fig 8A and Video 4S). However, with the rotation of Arg489 towards Glu962, the area becomes positively charged, which attracts the ligand from the water reservoir to form first interactions with Lys965 (Fig 8A). While Glu962 does not form direct interactions with the ligand (absent in the LRBN diagram, Fig 3C) it acts as a polarizable switch to effectively move the ligand from Lys965 to Arg489. Thus, once the ligand is passed to Arg489 the Arg489-Glu962 salt bridge breaks, while the Lys965-Glu962 salt bridge reforms.

With the ligand, Arg489 is now capable of moving to the active site. This movement, in turn, is facilitated by Glu463, Glu319, Glu497 and Glu519 of the active site, which attract the charged ammonium of the ligand (Fig 8B). In the final pose the ammonium group forms hydrogen bonds with Glu319, Glu463 and Glu519 in the active site, as it is observed in the available *Pf*M1-AAP crystal structures; a salt bridge with Arg489 in the active site is maintained in most of the trajectories. The direct interaction between the ligand and Arg489 is observed in one crystal structure (PDB code: 4X2U) [15], while in other crystal structures Arg489 is at the distance of water-mediated interactions with the ligand. From our sMD simulations, we found that Arg489 not only plays a role in stabilization of the ligands within the active site but also is involved in the ligand migration. The interaction with Arg489 is responsible for the third high-occupancy state in Fig 3B where Arg489 is highlighted as a residue forming strong and prolonged interactions with the ligands in the LRBN plot (Fig 3C).

### Product migration and release from the N-terminal channel

The crystal structure of P*f*M1-AAP bound to the Arg product was used as a starting point to study the product migration and release from the N-terminal channel of P*f*M1-AAP using the 30 ns and 100 ns sMD simulations.

From the proposed catalytic mechanism of the peptide bond cleavage in *Pf*M1-AAP [13,16], the carboxyl group of the amino acid product is deprotonated. The COO- anion of the amino acid product is stabilized by the Zn^+2^ ion and the OH-group of Tyr381 in the transition state, while the protonated amine product is released from the active site. This mechanism is supported by the available crystal structure of PfM1-AAP bound to the Arg product [17]. The carboxyl anion of Arg is bound to the Zn^+2^ and the OH group of Tyr381, whereas the protonated amino group of Arg is coordinated by three glutamate residues. We, therefore, use the ionized from of Arg in our simulations.

**Fig 9:**
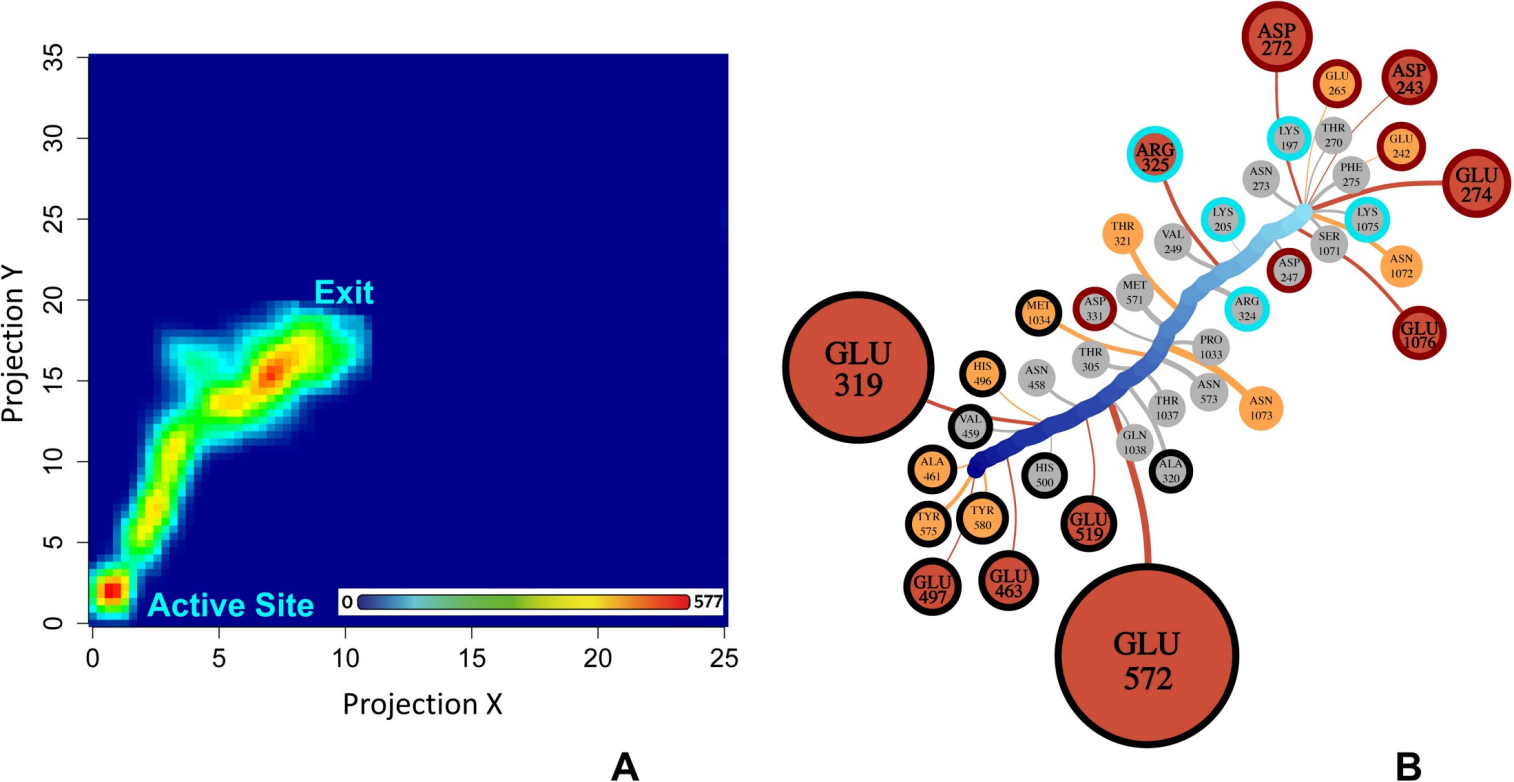
Ligand migration through the N-terminal channel. **A**: The 2D ligand occupancy map from the all 30-ns sMD trajectories of the Arg product migration plotted on the cross section through the channel centre (See in the Supporting Information, S1 Fig). The exit from the N-terminal channel and the active site are labelled. Red means high occupancy and blue low occupancy. Colouring is linear. **B**: The ligand-residue binding network (LRBN) derived from the ligand-residue interaction energy of the all sMD trajectories of the Arg product migration. Nodes are residues interacting with the ligand during the migration. The size and colour of the nodes correspond to the strength of interaction. The 25%, 50% strongest interactions and the remaining frequently occurring interactions are in red, yellow and grey, respectively. Edges reflect the interval of occurrence of a ligand-residue interaction; thicker edges show prolonged interactions. The average ligand path is shown as a thick light-to-dark-blue-changing colour line. The active site, positively-charged and negatively-charged residues are circled in black, blue and red, respectively.

The Arg occupancy plot shown in Fig 9A reveals an almost continuous narrow high-populated area throughout the entire unbinding pathway. The 2D occupancy map is plotted into the plane defined by the cross section through the N-terminal channel centre (S1 Fig). Although 184 residues of the N-terminal channel were considered in the construction of the LRBN diagram, only a couple of residues were identified as playing some role in the product egress (Fig 9B). Consequently, the high-populated area in the occupancy plot observed is due to sterically restrained migration rather than the formation of transient stable states by intermediate strong interactions with the enzyme. This is in correlation with a small R_gyr_ of the N-terminal channel and its notable deviation during the ligand migration (Table 1).

**Fig 10:**
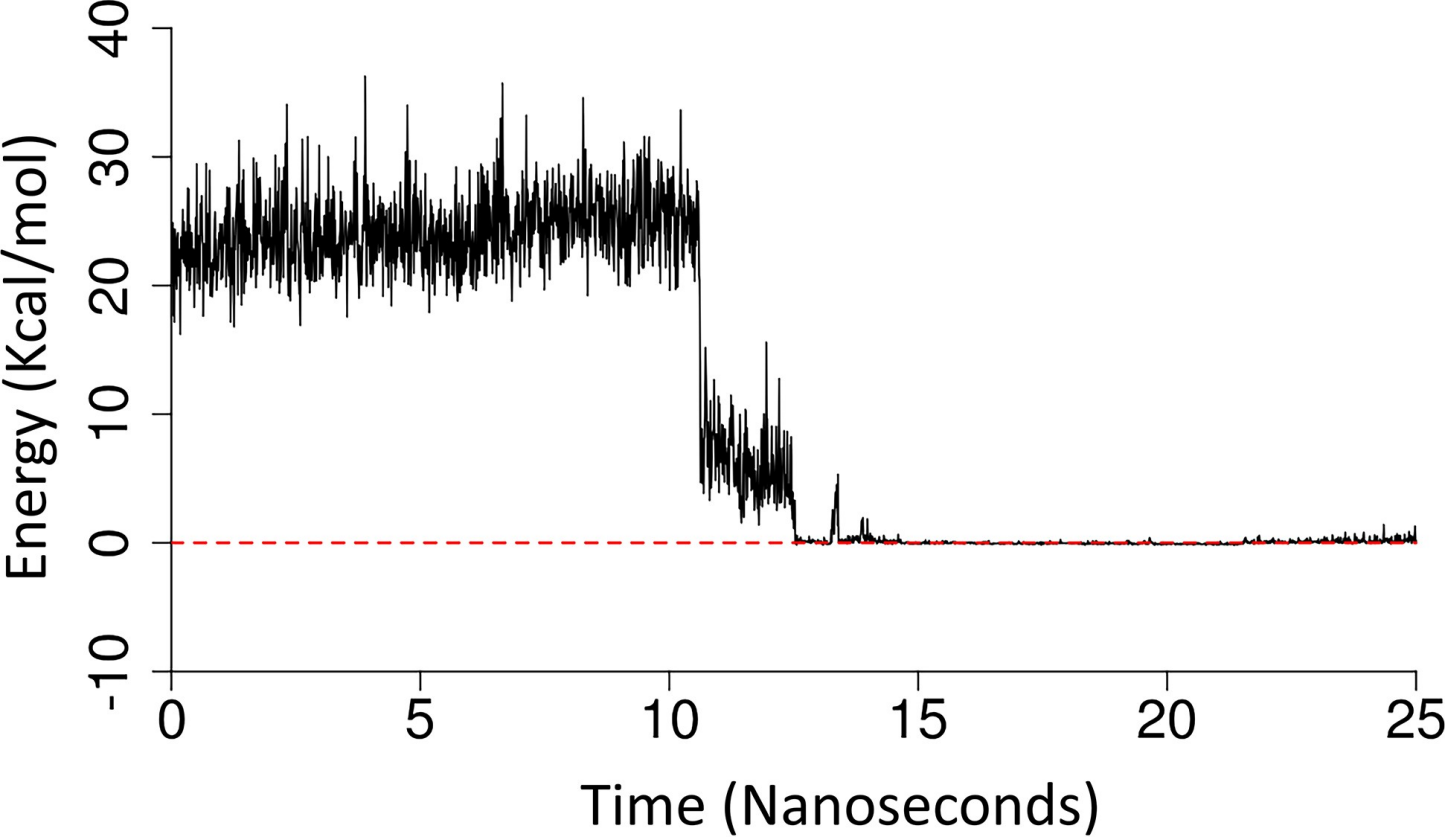
The non-bonded interaction energy between the carboxyl group of the Arg product and the glutamic acid bundle, involving Glu319, Glu463, Glu497 and Glu519 along the representative sMD trajectory.

As mentioned above, the active site of P*f*M1-AAP has four negatively charged glutamate residues (Glu319, Glu463, Glu497 and Glu519) that interact with the ammonium of the substrate. Following the substrate cleavage these polar interactions are severed, placing the carboxylic group of the product within a highly repulsive negatively charged environment. Fig 10 represents the sum of the non-bonded interaction energy between the carboxyl group of the Arg product and the glutamic acid bundle along the representative sMD trajectory, quantifying the strong, albeit brief, repulsive force of +24 kcal/mol. This repulsion helps to quickly propel the product out of the active site by breaking the interactions between the Arg product and the active site, and inducing the conformational changes required for the successful migration through the N-terminal channel.

**Fig 11:**
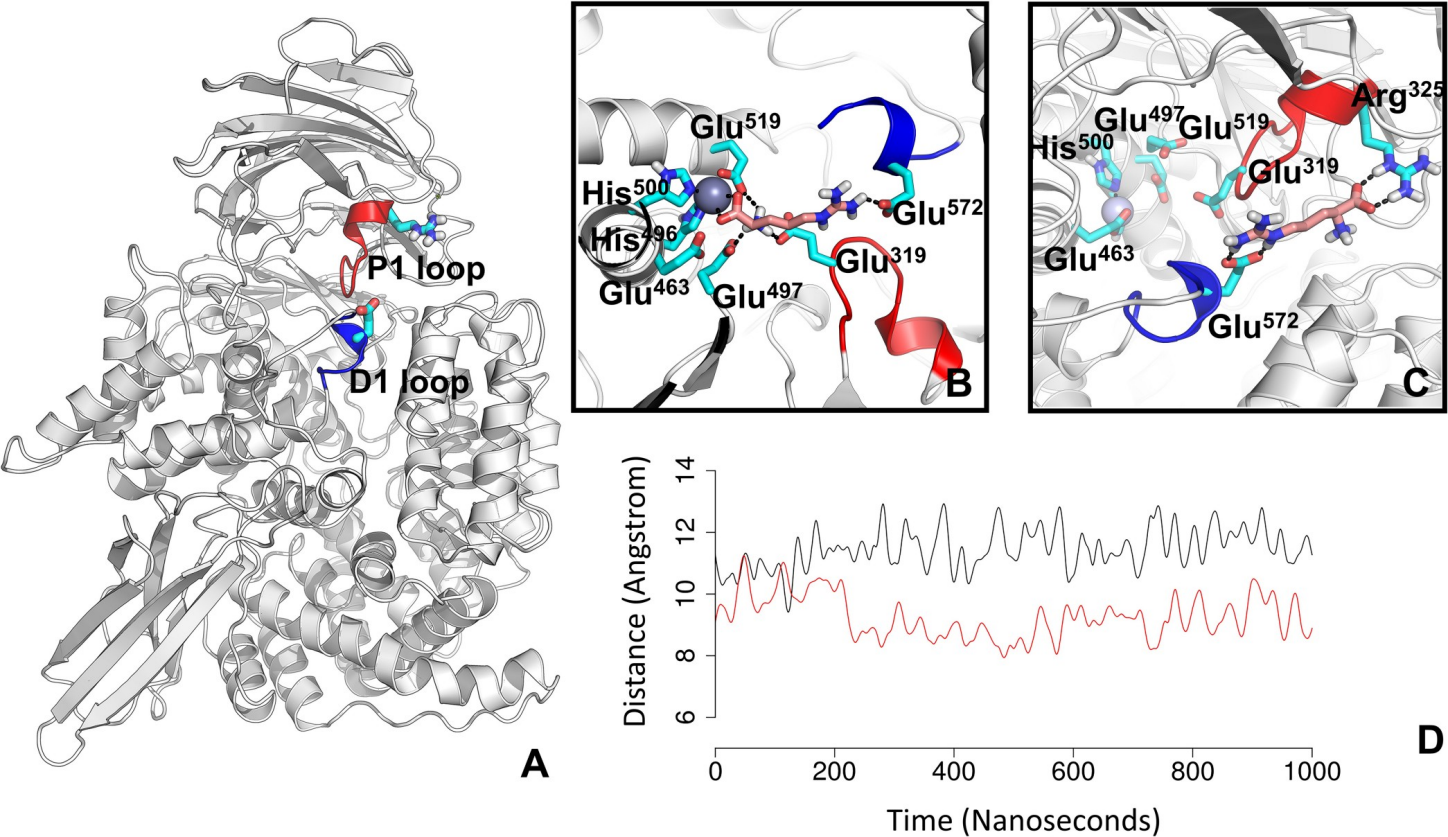
The opening of the N-terminal channel, involving loops D1 and P1. **A:** the overall structure of P*f*M1-AAP with highlighted loops; **B:** the Arg product bound to the active site; **C:** The interaction of Arg with Arg325 and Glu572 during the release from the active site. The salt bridges and hydrogen bonds are shown in a black-dotted line. **D:** The distance between the centre of mass of P1 and D1 loops in the1μs cMD simulations of the P*f*M1-AAP the ligand-unbound (red) and ligand-bound forms (black).

The opening of the N-terminal channel is encompassed by a protruding loop of domain II consisting of residues 570–575, referred to as the P1 pocket loop, and a loop of domain I, residue 317–325, referred to as the D1 loop (Fig 11A). Our sMD simulations indicate that the guanidine group of the product forms interactions with Glu572 of the P1 loop and the carboxyl group of the product forms temporal interactions with Arg325 of the D1 loop (Fig 11B and 11C). Interestingly, the 1μs cMD simulation of P*f*M1-AAP in the ligand-unbound form shows a salt bridge between Glu572 and Arg325, which is present in 90% of simulations. Calculation of the distance between the centre of mass of P1 and D1 loops shows that the loops are at the distance of 9 Å in the unbound form and 11.5 Å in the product-bound form of P*f*M1-AAP (Fig 11D). It is apparent that the Glu572-Arg325 interaction holds the loops together closing the access to the N-terminal channel, whereas in the absence of the salt bridge the loops are in an open position facilitating the egress of the product. The closed state of the N-terminal channel may prevent the substrates from prematurely exiting from the buried active site, while only when hydrolytic cleavage occurs the repulsive force separates the loops and allows the product to leave into the external environment (the parasitic cytosol).

## Discussion

To understand the physico-chemical properties between P*f*M1-AAP and its substrates/inhibitors, we carried out the multiple sMD simulation runs of five ligand-protein complexes using two simulation protocols totalling 2 μs simulation time and explored the atomistic detail of ligand migration. We then developed an efficient analysis of the MD trajectories to derive the common patterns of interactions along the binding channels from this large set of simulated data. Thus, by computing a ligand-residue interaction energy and ranking of the average interaction energy from the generated sMD trajectories, we have built a ligand-residue binding network that represents a general map of interactions during the ligand migration to and from the active site. This network was a key tool in our study to identify the critical residues in the ligand migration, which were then visually scrutinized to uncover their true function in a specific manner.

Our sMD simulation analysis has revealed a non-diffusive binding profile and identified several well-defined steps of ligand migration along the C-terminal channel, in particular: the ligand recognition at the channel entrance and the initial migration into the channel cavity (I), the ionic translation of the ligand to the water reservoir (II), the ligand reorientation in the water reservoir (III) and the final passage in a suitable conformation to the active site (IV). Several charged residues have been found subsequently interacting and pulling the ligands along the C-terminal channel pathway. In particular, we have identified two pairs of residues, Glu850/Lys907 and Asp830/Lys849, which coordinate the recognition and initial migration. Among them Glu850 forms the first strongest interaction with the enzyme, thus serving as a recognition point. Next, Arg969 is predicted to act as an ionic substrate translator, which facilitates the movement of the ligand to the water reservoir. The distinct conformations of this residue within the C-terminal channel that allow it to move the ligand a long distance have also been observed from the available crystal structures and the cMD simulations, validating our sMD findings. Finally, we identify Arg489 that controls the passage of the substrate in the suitable orientation to the active site. Overall, the extensive sMD simulations demonstrate that electrostatic interactions play a primary role in controlling the substrate and inhibitor migration in the C-terminal channel. To examine the conservation of these residues we have built the sequence alignment of P*f*M1-AAP from the 13 malaria species. We found that the residues are conserved or substituted with a residue of a similar charge (S2 Fig), suggesting a common mechanism of ligand migration in the M1-AAP of malaria species.

Measurement of P*f*M1-AAP activity in the presence of the haemoglobin-derived L-peptide and E-peptide provides a level of validation for the proposed ligand migration mechanism. In particular, there is a recognition and specificity spot at the entrance of the C-terminal channel, likely served by Glu850, which allows the L-peptide to enter and not the negatively-charged E-peptide. In addition, replacement of Arg969 with an Ala reduces the L-peptide binding, indicating its importance in migration of small ligands. Finally, although the L-peptide binds to the C-terminal channel, it is not cleaved in the active site because it either cannot reach the active site or is not in the suitable orientation to fit to the cleavage site. Thus, the ligand reorientation in the water reservoir is critical for binding to the active site and the large peptide cannot flip and turn in the small water cavity to form a suitable configuration for the active site. This also explains specificity of P*f*M1-AAP to cleave short (di and three)-peptides.

In all the simulations, the C-and N-terminal channels were fully hydrated. We consistently observe water-mediated hydrogen bonds (from one up to 5 contacts) between the ligand and enzyme during the ligand migration along the channels. The water molecules effectively substitute the contacts occurring between the ligands and the charged residues of the channel allowing a quick migration of the ligand toward the active site. In addition, the ability of water to shield long-range electrostatic interactions may prevent ligands from drifting back to the previous step. The water reservoir, which permits the formation of a water solvation shell around the ligand, assists the ligand to adapt a favourable conformation to best complement the active site.

From the sMD simulations, the sMD pulling of the Arg product along the N-terminal channel is likely accompanied by the repulsion force that pushes the product out from the active site and unlocks the external opening of the channel by breaking the Glu572-Arg325 interactions. These residues are also conserved among malaria species (S2 Fig), suggesting commonalities in the product release. The importance of Glu572 in the substrate binding and inhibitor specificity has been shown in the recent mutagenesis study [18].

Malaria *Pf*M1-AAP has been a challenging target for the development of active site inhibitors; for example, high through-put screening of a >200,000 chemical library did not identify inhibitors with IC50s below 10 uM [19]. The discovered atomistic description of the complex binding process in P*f*M1-AAP here provides a possible clue to the failure of random screenings and suggests opportunities to design a novel class of antimalarial agents in a rational way. The significance of the identified charged residues in the C-terminal channel can now be further explored by mutagenesis studies. In addition, the intermediate states stabilized by the interactions with the channel residues identified in the C-terminal channel can be probed as binding sites for small molecule ligands that could block or allosterically modulate P*f*M1-AAP. Notably, examination of the available crystal structures shows the regular presence of a glycerol molecule around Arg489 and Arg969, further supporting our hypothesis of the existence of the alternative binding sites in the C-terminal channel. This work also provides a structural foundation for future optimization of inhibitor-enzyme binding and unbinding rates.

## Conclusion

Our computer simulations further suggest that substrates and inhibitors enter the P*f*M1-AAP via the long C-terminal channel and for products to exit via the short N-terminal channel. We show that the migration of substrates through the C-terminal channel is predominantly controlled via long-range electrostatic interactions. Side chain rotations of key positively charged residues regulate the migration of ligands between the residues, endorsed by competitive enzymatic studies using peptides. The water reservoir in the core of P*f*M1-AAP is important for reorientation of substrates to fit to the cleavage site. The ligand-residue binding network tool developed here for an efficient analysis of the replicate sMD simulations can be employed in a wide variety of situations to identify and rank the critical residues of a protein interacting with other binding partners. The results presented in this study will facilitate design of efficient malarial M1 aminopeptidase inhibitors, helping in the race for new antimalarial drugs.

## Materials and methods

### Biosystem preparation

The initial coordinates for P*f*M1-AAP co-crystallized with the ligands Bestatin, R5X and Arg were acquired from the Protein Data Bank (PDB) repository (PDB ID: 3EBH, 4R5X and 4J3B) [9,17,20]. The Met-Phe and Arg-Ala di-peptides were created using the Maestro building tool [21] and docked into the active site (PDB ID: 3EBH) using the Induced fit protocol, version 6.6 [21]. The crystal structures were prepared with the Maestro protein preparation module [21]. The missing side chains were inserted using Prime version 3.8 ]21]. Overlapping hydrogen atoms were refined by hydrogen-only minimization and residues with alternate positions were defined. The engineered mutations (7 within 3EBH & 4R5X and 1 in 4J3B) were reversed to the wild type sequence. To alleviate the protein strain arising from these point mutations a two-stage minimization protocol was applied, i.e. hydrogen only minimization and all atom minimization with variable constraints set from the X-ray derived B factors in order to reduce the deviation from the original crystal structure coordinates. Minimization was performed using the MacroModel module [21]. The biosystems were solvated using TIP3P water molecules and 0.15 M Na^+^ and Cl^-^ ions in an orthorhombic box of 12 Å size using the System Builder of Maestro GUI [21].

### Molecular dynamics simulations

To fully equilibrate the five systems the following 6 step protocol was implemented: (1) simulation in the NVT ensemble with Brownian dynamics at 10 K for 120 ps with small time steps and solute non-hydrogen atoms restrained; (2) simulation using a Berendsen thermostat for 12 ps at the 10 K velocity re-sampling every 1 ps, a fast temperature relaxation constant and non-hydrogen solute atoms restrained; (3) simulation in the NPT ensemble using a Berendsen thermostat and barostat for 12 ps at 10 K and 1 atm, velocity resampling every 1ps a fast temperature relaxation constant, a slow pressure relaxation constant and non-hydrogen solute atoms restrained; (4) solvation of the protein cavity using the solvate pocket script; (5) simulation in the NPT ensemble using a Berendsen thermostat and barostat for 12 ps at 300 K and 1 atm with a fast temperature relaxation constant, a slow pressure relaxation constant velocity resampling every 1 ps and non-hydrogen solute atoms restrained; (6) simulation in the NPT ensemble using a Berendsen thermostat and barostat for 24 ps at 300 K and 1 atm with a fast temperature relaxation constant and a normal pressure relaxation constant. Each system was then subjected to a 70 ns MD simulation with no constraints applied.

Simulation conditions were maintained at 300 K constant temperature by Langevin dynamics and 1 atm constant pressure using the Nose Hoover Langevin piston method and the NPT ensemble. Long-range electrostatic interactions were calculated using the particle mesh Ewald method [22]. The catalytic Zn^+2^ ion with the pentahedral coordination was used in the simulations (13). A harmonic restraint was applied to the Zn^+2^ ion throughout all simulations with a force constant of 10 kcal/mol/Å^2^ to maintain the coordination with the active site residues. The RMSD of the Zn^+2^ ion from the crystal structure position was 1.5±0.3 Å during simulations, indicating the stability of the Zn^+2^ ion. The Zn^+2^ ion was at the distance of 2.3±0.1, 2.2±0.1 and 2.0±0.04 Å with the coordinating residues: His496, His500 and Glu519, respectively, during the simulations. The Zn^+2^ ion coordinates with a water molecule and the ligand. In the unbinding event, the ligand coordination was broken and substituted with a second water molecule.

sMD simulations were set up and carried out using the Desmond source distribution v3.6.1.1 [23] with the OPLS-2005 [24,25] force field and the biasing force plugin. A biasing potential was applied as a time-dependent harmonic spring. Here, atoms of the ligand are restrained with respect to the atoms of the protein. In this way, a force, which is distributed based on the centre of mass of the ligand and protein atoms, steers the ligand along a specified vector that is determined as a line through the centroid of the ligand and protein atoms. Steering was performed using a pulling velocity of 0.015 Å/ps and a biasing force constant of 10 kcal/mol/Å^2^ in the 30 ns simulation runs. The pulling velocity was reduced to 0.0045 Å/ps in the 100 ns simulation runs. This protocol represents the optimal pulling velocity and biasing force constant out of the following tested combinations: 0.030, 0.025, 0.020, 0.015, 0.010, 0.005 Å/ps and each with the force constant of 2.5, 5.0, 7.5 and 10.0 kcal/mol/Å^2^. The input velocities are determined by the Maxwell-Boltzmann distribution at 300 K.

The 1μs classical MD simulations of P*f*M1-AAP in the ligand-bound and unbound forms (PDB ID: 4J3B and 3EBG) [9,17] were performed. The buffer of water surrounding the protein was increased up to 15 Å. The distance for the short-range component of the electrostatic calculations was increased using a tapering function from 9 to 12 Å with the long-range electrostatic forces calculated every 4 fs (reduced from 6 fs). The harmonic restraint on the zinc ion was kept to preserve the pentahedral coordination.

### Trajectory analyses

R_gyr_ based on selection of the Cα atoms was calculated around the C-terminal channel opening using residues 972, 938, 907, 850, 846 and 1008 (1); around Arg969 selecting residues 969, 829, 900, 934, 1006 and 826 (2); around the water reservoir using 889, 484, 483, 965, 538, 550 (3) and 1042; and around Arg489 using 489, 581, 1038 and 997 (4) using VMD v1.9.2 (26). In the case of the N-terminal channel, R_gyr_ was calculated in the internal opening selecting residues 320, 305, 575 and 1034 (1); and in the external opening selecting residues 249, 570 and 1073 (2). In the sMD simulations, only frames, where the ligand sits around the selected residues were considered for R_gyr_ calculation to identify the maximum of R_gyr_ value variation.

The work required to pull ligands from the active sites was calculated by integrating the force over the ligand travelled distance using the VMD script [26] and the *in house* R script [27].

The ligand occupancy maps were computed using the *in house* R script [27]. Initially, all the trajectories were aligned based on the Cα atoms of the protein. The centre of mass (COM) of the ligand was used to derive a positional map for each ligand. The 3D coordinates of the COM of all the ligands were translated to the 2D coordinates using the plane defined by the cross section through the centre of either the C-terminal channel or the N-terminal channel. Next, the 2D coordinates of the ligand COM were used to create a heatmap. The generated graphs show the general binding path and the area sampled by the ligands. The VolMap plugin of VMD v.19.2 [26] was used to show the plane and 2D volume slice in Fig 3A and S1 Fig defined by the cross section through the centre of the channel.

For construction of the LRBN diagrams the ligand non-bonded interaction energy for each residue of the channels was calculated using the ‘analyze_trajectories.py’ script from the Desmond tools [23]. Long-range electrostatics was calculated using the Particle Mesh Ewald method with a cutoff value of 9 Å [22]. The OPLS-2005 force field [24,25] was used. The residues lining the N- and C-terminal channels were determined from a representative sMD simulation of each ligand, where those residues that fall within 4 Å of the ligand as it traverses the channel are selected. A visual inspection of the selection was made to ensure all residues encompassing the channel are included. We selected 214 and 184 residues of the C-terminal and N-terminal channels, respectivly. The LRBN diagrams were computed with the *in house* R script [27] and visualized with the igraph package [28]. The LRBN aggregates the results of ligand-residue interaction energies for all the simulations and visualizes as an ensemble average in the form of nodes (residues) and edges (the timeframe of residue-ligand interaction). A 2D representation of the ligand average path is derived from the COM of all the ligands. Nodes connect to this path at the point they have the strongest interaction energy. The ligand-residue interaction is counted at the distance of 9 Å. Nodes are ranked into three groups based on the percentile ranking, less than 0.5, between 0.5 and 0.75 and greater than 0.75.

The images were rendered in PyMol v2.0 [21] and VMD v1.9.2 [26]. The graphs of RMSF and ligand occupancies in water were calculated in VMD v1.9.2 and plotted using the R program [27]. The water-mediated occupancy was calculated in VMD using the Tk/Tcl interpreter. The sequence alignment of the M1 aminopeptidase from different malaria species was performed using ClustalX [29]. The videos 1S-2S and 4S were rendered in PyMol v2.0 [21] and video 3S in VMD v1.9.2 [26].

### Enzymatic studies

Functionally active recombinant wild-type P*f*M1-AAP and the variant PfM1-AAP Arg969Ala were expressed in *Escherichia coli* and purified by affinity chromatography on Ni-NTA columns as described by McGowan et al. [9]. Enzymatic assays were performed using the fluorogenic peptide substrate H-Arg-7-amido-4-methyl courmarin (H-Arg-NHMec) at a concentration of 5 μM in Tris HCl, pH 7.5 at 37°C. After initiating the enzymatic reaction, activity was recorded as relative fluorescent units in a fluorometre with excitation set at 370 nm and emission at 460 nm. The haemoglobin-derived peptides, L-SFPTTK (L-peptide) and E-EKSAVTA (E-peptide), were synthesised by GL Biochem, Shanghai, China, and dissolved in water before use.

## Supporting information

S1 Video Ligand recognition by Glu850, Lys849, Lys849 and Asp830. (MP4)

S2 Video Arg969 acts as an ionic regulator. (MP4)

S3 Video Ligand flipping in the water reservoir. (MP4)

S4 Video Entry to the active site. (MP4)

S1 Table Simulation systems and details of sMD simulations. (DOCX)

S2 Table Fluctuation of the radius of gyration at several locations of the C- and N-terminal channels in the cMD simulations of the empty *Pf*M1-AAP and during the ligand passage in the 30ns sMD simulations. (DOCX)

S1 Fig Definition of 2D projection to create a ligand occupancy map. The plane defined by the cross section through either the centre of the C-terminal channel (A) or the centre of the N-terminal channel (B) was used to map the 3D coordinates of the centre of mass (COM) of all the ligands into the X and Y projections. The plane is in blue and the protein slice in the plane is in red.
(DOCX)

S2 Fig The alignment of M1 aminopeptidase sequences from malaria strains. Residues involved in the migration of the ligands from the C-terminal channel are highlighted in cyan. Glu572 and Arg325 forming a salt bridge are in pink.
(DOCX)

S1 File 30ns MD simulation parameters and configuration files for the C-terminal channel. Unzipping this file will create a directory containing the parameters and starting structures used for the 30ns sMD simulations.
(ZIP)

S2 File 100ns MD simulation parameters and configuration files for the C-terminal channel. Unzipping this file will create a directory containing the parameters and starting structures used for the 100ns sMD simulations.
(ZIP)

S3 File 30ns MD simulation parameters and configuration files for the N-terminal channel. Unzipping this file will create a directory containing the parameters and starting structures used for the 30ns sMD simulations.
(ZIP)

S4 File 100ns MD simulation parameters and configuration files for the N-terminal channel. Unzipping this file will create a directory containing the parameters and starting structures used for the 100ns sMD simulations.
(ZIP)

S1 Link Ligand-Residue Binding Network Script is located here: https://github.com/Danny221212/sMD-PF-M1AAP. (DOCX)
