# Supplementary material for: Steered molecular dynamics simulations reveal critical residues for (un)binding of substrates, inhibitors and a product to the malarial M1 aminopeptidase
Source: PLoS Comput Biol. 2018 Oct 31;14(10):e1006525. doi: 10.1371/journal.pcbi.1006525 (PMC6239339; doi:10.1371/journal.pcbi.1006525)
Supplement: S1 Table — (DOCX) [file pcbi.1006525.s005.docx]

| *PfM1*-AAP Biosystem | Simulation Time | Pulling Velocity | Force Constant | Simulation Count | |
| --- | --- | --- | --- | --- | --- |
| Ligands | [ nanoseconds ] | [ Å/ps ] | [ kcal/mol ] | N-Terminal | C-Terminal |
| Arg | 30 | 0.0015 | 10 | 6 | 6 |
|  | 100 | 0.00045 | 10 | 1 | 1 |
| Arg-Ala | 30 | 0.0015 | 10 | 6 | 6 |
|  | 100 | 0.00045 | 10 | 1 | 1 |
| Met-Phe | 30 | 0.0015 | 10 | 6 | 6 |
|  | 100 | 0.00045 | 10 | 1 | 1 |
| Bestatin | 30 | 0.0015 | 10 | 6 | 6 |
|  | 100 | 0.00045 | 10 | 1 | 1 |
| R5X | 30 | 0.0015 | 10 | 6 | 6 |
|  | 100 | 0.00045 | 10 | 1 | 1 |

**Table 1S.** Simulation systems and details of sMD simulations.
