# Supplementary material for: Steered molecular dynamics simulations reveal critical residues for (un)binding of substrates, inhibitors and a product to the malarial M1 aminopeptidase
Source: PLoS Comput Biol. 2018 Oct 31;14(10):e1006525. doi: 10.1371/journal.pcbi.1006525 (PMC6239339; doi:10.1371/journal.pcbi.1006525)
Supplement: S2 Table — (DOCX) [file pcbi.1006525.s006.docx]

| *Pf*M1-AAP Systems | **C-terminal Channel** | | | | | | | | **N-terminal Channel** | | | |
| --- | --- | --- | --- | --- | --- | --- | --- | --- | --- | --- | --- | --- |
|  | Opening | | Around Arg969 | | Around water reservoir | | Around Arg489 | | Internal Opening | | External Opening | |
|  | R_gyr_, Å | Δ, Å | R_gyr_, Å | Δ, Å | R_gyr,_ Å | Δ, Å | R_gyr,_ Å | Δ, Å | R_gyr,_ Å | Δ, Å | R_gyr,_ Å | Δ, Å |
| empty | 8.9±0.2 |  | 8.6±0.1 |  | 12.7±0.2 |  | 9.7±0.1 |  | 5.9±0.1 |  | 6.9±0.2 |  |
| Arg-Ala | 8.9±0.4 | 0.1 | 9.2±0.4 | 0.6 | 13.4±0.3 | 0.7 | 10.8±0.4 | 1.1 | 8.1±0.4 | 2.1 | 9.1±0.6 | 2.2 |
| Met-Phe | 9.3±0.5 | 0.4 | 8.9±0.5 | 0.3 | 13.0±0.3 | 0.3 | 10.1±0.3 | 0.4 | 7.8±0.4 | 1.9 | 8.4±0.7 | 1.5 |
| Bestatin | 8.9±0.4 | 0.1 | 8.7±0.4 | 0.2 | 13.1±0.3 | 0.4 | 10.2±0.3 | 0.5 | 7.7±0.4 | 1.7 | 8.3±0.6 | 1.4 |
| R5X | 8.8±0.4 | 0.1 | 9.1±0.4 | 0.6 | 13.0±0.3 | 0.3 | 10.5±0.3 | 0.8 | 8.1±0.3 | 2.1 | 7.9±0.5 | 1.0 |
| Arg | 8.3±0.3 | 0.6 | 8.3±0.3 | 0.2 | 13.2±0.3 | 0.5 | 10.4±0.3 | 0.7 | 7.4±0.4 | 1.4 | 8.3±0.5 | 1.4 |

**Table 2S**. Fluctuation of the radius of gyration at several locations of the C- and N-terminal channels in the cMD simulations of the empty *Pf*M1-AAP and during the ligand passage in the 30ns sMD simulations. Definition of the radius of gyration is described in the methods.
