# Supplementary material for: Steered molecular dynamics simulations reveal critical residues for (un)binding of substrates, inhibitors and a product to the malarial M1 aminopeptidase
Source: PLoS Comput Biol. 2018 Oct 31;14(10):e1006525. doi: 10.1371/journal.pcbi.1006525 (PMC6239339; doi:10.1371/journal.pcbi.1006525)
Supplement: S1 Fig — The plane defined by the cross section through either the centre of the C-terminal channel (A) or the centre of the N-terminal channel (B) was used to map the 3D coordinates of the centre of mass (COM) of all the ligands into the X and Y projections. The plane is in blue and the protein slice in the plane is in red. (DOCX) [file pcbi.1006525.s007.docx]

**
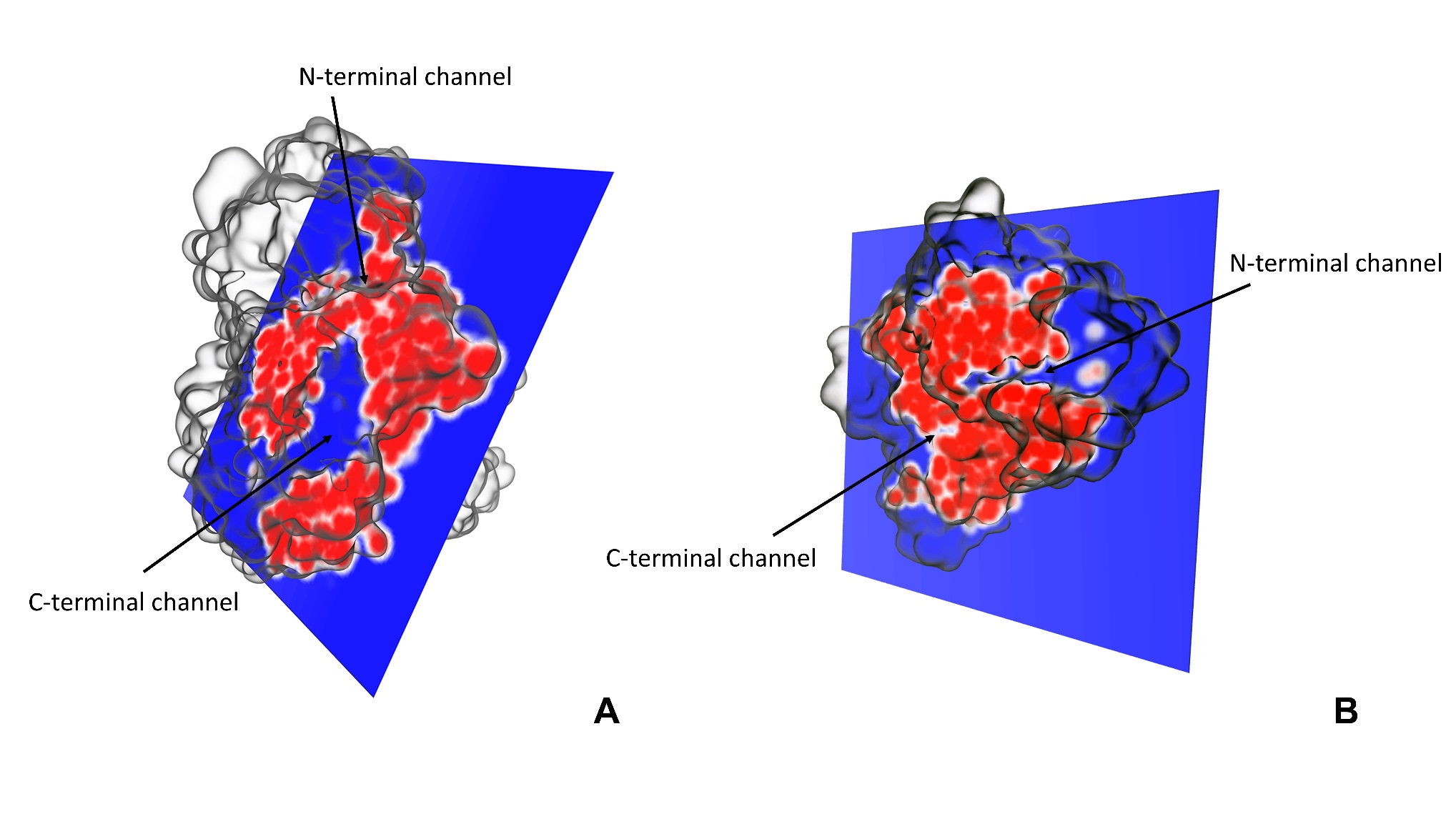
**

**Figure 1S:** Definition of 2D projection to create a ligand occupancy map. The plane defined by the cross section through either the centre of the C-terminal channel (A) or the centre of the N-terminal channel (B) was used to map the 3D coordinates of the centre of mass (COM) of all the ligands into the X and Y projections. The plane is in blue and the protein slice in the plane is in red.
