# Supplementary material for: Steered molecular dynamics simulations reveal critical residues for (un)binding of substrates, inhibitors and a product to the malarial M1 aminopeptidase
Source: PLoS Comput Biol. 2018 Oct 31;14(10):e1006525. doi: 10.1371/journal.pcbi.1006525 (PMC6239339; doi:10.1371/journal.pcbi.1006525)
Supplement: S2 Fig — Residues involved in the migration of the ligands from the C-terminal channel are highlighted in cyan. Glu572 and Arg325 forming a salt bridge are in pink. (DOCX) [file pcbi.1006525.s008.docx]

* 20 * 40 * 60 *
3EBG_A|PDB : ---------------------------------------------------------------------- : -
Pl_falcipa : MKLTKGCAYKYIIFTVLILANILYDNKKRCMIKKNLRISSCGIISRLLK---SNSNYNSFNKNYNFTSAI : 67
Pl_berghei : MVLKKLLCFNLFLIIILTFENLSFDKKNTCMINNTIRSNSCCIVNRVLR----EKTHHYSSKISKSIPFI : 66
Pl_yoelii : MVTKKLLSFNLFLIIILTFENLTFDKKNTCMINNTIRPNSCGIVNRVLR----EKPYHYSSKISKSIPFI : 66
Pl_chabaud : MVTKKLLSFNLFLIIVLTFENLSFVKKNTCMINNTIRPNSCGIVNRVLR----EKSQQHSSKIPKILPFI : 66
Pl_petteri : MVTKKLLSFNLFLIIILTFENLSFDKKNTCMINNTIRPNSCGIVNRVLR----EKPQNNSIKTPKILPFI : 66
Pl_vinckei : MVTKKLLSFNLFLIIILTFENLSFDKKNTCMINNNIRSNSCGIVNRMLR----ENSQNNSLKTPKILPYI : 66
Pl_cynomol : --------FLFVTVLVALANYTPFDYQNTCMISKSCRKNSCGIISRVLSGIHVNKTSARAKALISLSSLI : 62
Pl_vivax : ------FHFLFITVLVALANYTPVDYQNTCMISKSCRKNSCGITSRVLSGIHVNKTSARAKALISLSSLI : 64
Pl_knowles : -------YFLFITVLAALAIYTPVDYQNTCMISRSCRKNSCGITSRVLSGIHVNKASTRARALISLSSLI : 63
Pl_fragile : --------FLLITVLVALEKYTPVDYQNTCMISRSCRKNSCGNISRVLSGIHVNRTSTRARALLSLSSLI : 62
Pl_reichen : MKLTKGCAYKYIIFTVLILANILYDNKKRCMIKKNLRISSCGIISRLLK---SNSNYNSFNKNYNFTSAI : 67
Pl_gaboni : MKLRKGCAYKYIIFTVLILANILYDNKKRCMIKKNLRISSCGIISRLLK---SNSNYNSFNKNYNFTSAI : 67

80 * 100 * 120 * 140
3EBG_A|PDB : ---------------------------------------------------------------------- : -
Pl_falcipa : SELQFSNFWNLDILQKDIFS--NIHNNKNKPQSYIIHKRLMSEKGDNNNNN---HQNNNGNDNKKRLGSV : 132
Pl_berghei : QNFSLEKYFTGESLQKNILNNINKLGG------------------------------AHLFHISKSHLTA : 106
Pl_yoelii : QNFSLEKNFTGESLQKNILNNINKLGG------------------------------AHLFHISKSHLAA : 106
Pl_chabaud : RNFSLKKYITGDQLQKNILNHINKLSG------------------------------AHLFQGSKSQLSS : 106
Pl_petteri : RNFSLEKYIAGDQLQKNILNNINKLSG------------------------------AHLFRDSKSQLPV : 106
Pl_vinckei : RNFSLEKYIAGDKLQKNILNNINKLSG------------------------------AHLFHDSKSQLPT : 106
Pl_cynomol : YQLQLPKLVSLDLFRRDLFTGVKQKGKRSPVPSYIIQNRLMSENIDSGSTNMSVTANQDKKRPGTGDGSE : 132
Pl_vivax : YHLQLPKLVSLDLFRRDLFTGVKQKGKRSPVPSYIIQNRLMSENGDSGSTNMSVTANQEKKRPGTGDASE : 134
Pl_knowles : YQLQLPKLVSLDLFRRDLFTGVNQKGRRSPVPSYIIQSRLMSENIDSGNNNMSATGNQEKKRPVTGDASD : 133
Pl_fragile : YHLQLPKLVSLDLFRRDLFTGVNQKGRRNPVPSYIIQNRLMSENIDKGNTNMSAQGNQDKKRPGTVDASD : 132
Pl_reichen : SELQFSNFWNLDILQKDIFS--NIHNNKNKPQSYIIHKRLMSEKGDNNNNNNNNHQNNNGNDNKKRLGSV : 135
Pl_gaboni : SELQFSNFWNLDILQKDIFN--NIHNNKNKPQSYIIHKRLMSEKGDNHNNQ-----NNNGNDNKKRLGSV : 130

* 160 * 180 * 200 *
3EBG_A|PDB : ----------------------------------------------------------------PKIHYR : 6
Pl_falcipa : VNNEENTCSDKRMKPFEEGHGITQVDKMNNNS-DHLQQNGVMNLNSNNVENNNNNNSVVVKKNEPKIHYR : 201
Pl_berghei : KSGNKNTEFIGEATELFKGFKRNFGINMTENKQTNIGRMLCENDNNNGGEDTSTEKAIFKKSKDSQIHYR : 176
Pl_yoelii : KAGNKNTEFIGEATELFKGFKRNFGINMTENKQTNIGRMLCEDDNNNGGEVTSTEKTIFKNSKDPQIHYR : 176
Pl_chabaud : ITNNK---FVGDATKSALGFVSNIGTDISGNKKTNLGRMLCEGHNNNGGEVTSTENAILKNSKDPQIHYR : 173
Pl_petteri : ITNNK---FAGDATKSVLEFRRNFGTNITENKKANFGRMLCEDHKNNGGEVASTEKAIFKNAKDPQIHYR : 173
Pl_vinckei : KPNNK---FVGDATKSFLESRRNFGTNQIENKRTNFGRMLCEDHKNNGGEVASTEKAVFKNPKDPKIHYR : 173
Pl_cynomol : GNNQGGSSTSATDKRFKEMDKNEESSNVSGSTNATLTNGTNSTTDGGENNNNG---SGNQGKNEPTIHYR : 199
Pl_vivax : GNNQSGISAAAQDKRMKGGDQSEEVSNVSGSTNAAMTNGASSTTEGGDNNNNG---SGNDGKNEPKIHYR : 201
Pl_knowles : AKNPDGSTISAQDKRMKGIDQNEGTSSVSGSTNAAFTNGASSIMEGGENNNNG---TGNEGKNEPTIHYR : 200
Pl_fragile : ANNQGASGTCTQAKRVKGIDQNEQSSNVSGSTNAAFTSGVN-TIMGDDNNNNG---SGNDGKNEPKIHYR : 198
Pl_reichen : VNNEENTCSDKRMKPFEEGHGITQVDKMNSNSDHHLQQNGVMNLNTNNVENNN--NSAVVKKNEPKIHYR : 203
Pl_gaboni : VNNEENTCSDKRMKPFEESHGFTQVDKMNNNIDNLQQ-NGVMNLNSNNVDNNN---SVVAKKNEPKIHYR : 196

220 * 240 * 260 * 280
3EBG_A|PDB : KDYKPSGFIINQVTLNINIHDQETIVRSVLDMDISKHNVGEDLVFDGVGLKINEISINNKKLVEGEEYTY : 76
Pl_falcipa : KDYKPSGFIINNVTLNINIHDNETIVRSVLDMDISKHNVGEDLVFDGVGLKINEISINNKKLVEGEEYTY : 271
Pl_berghei : TDYKPSGFTIDNVTLNINIFDNETIVRSSLNMCTNENYADEDLVFDGVGLSIKEISINNNKLTEGEDYTY : 246
Pl_yoelii : TDYKPSGFTIDNVTLNINIFDNETIVRSSLNMCTNENYADEDLVFDGVGLSIKEISINNNKLNEGEDYTY : 246
Pl_chabaud : TDYKPSGFTIDTVTLNINIFDNETTVRSSLSMCTNDNYANEDLVFDGVGLSIKEININDNKLTEGEDYTY : 243
Pl_petteri : TDYKPSGFTIDTVTLNINIFDNETTVRSSLSMCTNDNYADEDLVFDGVGLSIKELSINDNKLTEGEDYTY : 243
Pl_vinckei : TDYKPSGFTIDTVTLNINIFDTETTVRSSLNMCTNDNYANEDLVFDGIGLSIKELSINDNKLTEGEDYTY : 243
Pl_cynomol : KDYKPSGFVIDNVTLNINIFDNETSVRSTLDMKLSEHYRGEDLIFDGVSLEIKEISIDGNKLKEGEHYKY : 269
Pl_vivax : KDYKPSGFVIDNVTLNINIFDNETSVRSTLDMKLSEHYGGEDLIFDGVSLEIKEISIDNNKLMEGEHYKY : 271
Pl_knowles : KDYRPSGFIIDNVTLNINIFDNETSVRSTLDMKLSDHYRGEDLIFDGVSLEIKEISIDGNKLMEGEHYKY : 270
Pl_fragile : TDYKPSGFVIDNVTLNINIFDNETSVRSNLDMKLSEHYKGEDLVFDGVSLEIKEISIDNNKLVQGEHYKY : 268
Pl_reichen : KDYKPSGFIINNVTLNINIHDNETIVRSILDMDISKHNVGEDLVFDGVGLKINEISIDNKKLVEGEEYTY : 273
Pl_gaboni : KDYKPSGFVINNVTLNINIHDNETTVRSILDMDISKHNAGEDLVFDGVGLKINEISINNKKLVEGEEYTY : 266

* 300 * 320 * 340 *
3EBG_A|PDB : DNEFLTIFSKFVPKSKFAFSSEVIIHPETNYALTGLYKSKNIIVSQCEATGFRRITFFIDRPDMMAKYDV : 146
Pl_falcipa : DNEFLTIFSKFVPKSKFAFSSEVIIHPETNYALTGLYKSKNIIVSQCEATGFRRITFFIDRPDMMAKYDV : 341
Pl_berghei : DNEFLTIFAKNVPKENFVFLSEVVIHPETNYALTGLYKSKDIIVSQCEATGFRRITFFIDRPDMMAKYDV : 316
Pl_yoelii : DNEFLTIFAKNVPKENFVFLSEVVIHPETNYALTGLYKSKDIIVSQCEATGFRRITFFIDRPDMMAKYDV : 316
Pl_chabaud : DNEFLTVFAKNVPKGNFVFVSEVIIHPETNYALTGLYKSKDIIVSQCEATGFRRITFFIDRPDMMAKYDV : 313
Pl_petteri : DNEFLTIFAKNVPKGNFVFVSEVIIHPETNYALTGLYKSKDIIVSQCEATGFRRITFFIDRPDMMAKYDV : 313
Pl_vinckei : DNEFLTIFAKNVPKGNFVFVSEVIIHPETNYALTGLYKSKDIIVSQCEATGFRRITFFIDRPDMMAKYDV : 313
Pl_cynomol : DNEFLTIYSKFIPKGKFTFGSEVIIHPETNYALTGLYKSKNIIVSQCEATGFRRITFFIDRPDMMAKYDV : 339
Pl_vivax : DNEFLTIYSKFIPKGKFTFGSEVIIHPETNYALTGLYKSKNIIVSQCEATGFRRITFFIDRPDMMAKYDV : 341
Pl_knowles : DKEFLTIYSKFIPKGKFTFGSEVIIHPETNYALTGLYKSKNIIVSQCEATGFRRITFFIDRPDMMAKYDV : 340
Pl_fragile : DNEFLTIYSKYIPKGKFTFASEVIIHPETNYALTGLYKSKNIIVSQCEATGFRRITFFIDRPDMMAKYDV : 338
Pl_reichen : DNEFLTIFSKFVPKSKFAFSSEVIIHPETNYALTGLYKSKNIIVSQCEATGFRRITFFIDRPDMMAKYDV : 343
Pl_gaboni : DNEFLTIFSKFVPKSKFAFSSEVIIHPETNYALTGLYKSKNIIVSQCEATGFRRITFFIDRPDMMAKYDV : 336
 **|**

**Arg325**

360 * 380 * 400 * 420
3EBG_A|PDB : TVTADKEKYPVLLSNGDKVNEFEIPGGRHGARFNDPPLKPCYLFAVVAGDLKHLSATYITKYTKKKVELY : 216
Pl_falcipa : TVTADKEKYPVLLSNGDKVNEFEIPGGRHGARFNDPHLKPCYLFAVVAGDLKHLSATYITKYTKKKVELY : 411
Pl_berghei : TLTADKKKYPVLLSNGDKLNEFDIPGGRHGARFNDPHLKPCYLFAVVAGDLKHLSDNYVTKYTKKPVELY : 386
Pl_yoelii : TLTADKTKYPVLLSNGDKLNEFDIPGGRHGARFNDPHLKPCYLFAVVAGDLKHLSDNYVTKYTKKPVELY : 386
Pl_chabaud : TLTADKTKYPVLLSNGDKLNEFDIPGGRHGARFNDPHLKPCYLFAVVAGDLKFLSDKYVTKFTKKPVELY : 383
Pl_petteri : TLTADKTKYPVLLSNGDKLNEFDIPGGRHGARFNDPHLKPCYLFAVVAGDLKFLSDKYVTKYTNKPVELY : 383
Pl_vinckei : TLTADKKKYPVLLSNGDKLNEFDVPGGRHGARFNDPHLKPCYLFAVVAGDLKFLSDKYVTKFTNKPVELY : 383
Pl_cynomol : TVTADKEKYPVLLSNGDKLNEFDIPGGRHGARFNDPHLKPCYLFAVVAGDLKHLSDNYVTKFSKKKVELY : 409
Pl_vivax : TITADKEKYPVLLSNGDKLNEFEIPGGRHGARFNDPYLKPCYLFAVVAGDLKHLSDNYVTKFSKKNVELY : 411
Pl_knowles : TVTADKEKYPVLLSNGDKLNEFEIPGGRHGARFNDPYLKPCYLFAVVAGDLKHLSDNYVTKFSKRNVELY : 410
Pl_fragile : TVTADKQKYPVLLSNGDKLNEFDIPGGRHGARFNDPHLKPCYLFAVVAGDLKHLSDKYVTKFSKRNVELY : 408
Pl_reichen : TVTADKEKYPVLLSNGDKVNEFEIAGGRHGARFNDPHLKPCYLFAVVAGDLKHLSATYITKYTKKKVELY : 413
Pl_gaboni : TVTADKQKYPVLLSNGDKVNEFDIPGGRHGARFNDPHLKPCYLFAVVAGDLKHLSATYTTKYTKKKIELY : 406

* 440 * 460 * 480 *
3EBG_A|PDB : VFSEEKYVSKLQWALECLKKSMAFDEDYFGLEYDLSRLNLVAVSDFNVGAMENKGLNIFNANSLLASKKN : 286
Pl_falcipa : VFSEEKYVSKLQWALECLKKSMAFDEDYFGLEYDLSRLNLVAVSDFNVGAMENKGLNIFNANSLLASKKN : 481
Pl_berghei : VYSEAKYVSKLKWALECLKKAMKFDEDYFGLEYDLSRLNLVAVSDFNVGAMENKGLNIFNADSLLASKKT : 456
Pl_yoelii : VYSEAKYVSKLKWALECLKKAMKFDEDYFGLEYDLSRLNLVAVSDFNVGAMENKGLNIFNADSLLASKKT : 456
Pl_chabaud : VYSEEKYVSKLKWALECLKKAMKFDEDYFGLEYDLSRLNLVAVSDFNVGAMENKGLNIFNADSLLASKKT : 453
Pl_petteri : VYSEEKYVSKLKWALECLKKAMKFDEDYFGLEYDLSRLNLVAVSDFNVGAMENKGLNIFNADSLLASKKN : 453
Pl_vinckei : VYSEAKYVSKLKWALECLKKAMKFDEDYFGLEYDLSRLNLVAVSDFNVGAMENKGLNIFNADSLLASKKT : 453
Pl_cynomol : VFSEEKYVSKLKWALECLKKAMKFDEDYFGLEYDLSRLNLVAVSDFNVGAMENKGLNIFNANSLLASKKK : 479
Pl_vivax : VFSEEKYVSKLKWALECLKKAMKFDEDYFGLEYDLSRLNLVAVSDFNVGAMENKGLNIFNANSLLASKKK : 481
Pl_knowles : VFSEEKYVSKLKWALECLKKAMKFDEDYFGLEYDLSRLNLVAVSDFNVGAMENKGLNIFNANSLLASKKK : 480
Pl_fragile : VFSEEKYVSKLKWALECLKKAMKFDEDYFGLEYDLSRLNLVAVSDFNVGAMENKGLNIFNANSLLASKKK : 478
Pl_reichen : VFSEEKYVSKLQWALECLKKSMAFDEDYFGLEYDLSRLNLVAVSDFNVGAMENKGLNIFNANSLLASKKN : 483
Pl_gaboni : VFSEEKYVSKLQWALECLKKSMAFDEDYFGLEYDLSRLNLVAVSDFNVGAMENKGLNIFNANSLLASKKN : 476

500 * 520 * 540 * 560
3EBG_A|PDB : SIDFSYARILTVVGHEYFHQYTGNRVTLRDWFQLTLKEGLTVHRENLFSEEMTKTVTTRLSHVDLLRSVQ : 356
Pl_falcipa : SIDFSYARILTVVGHEYFHNYTGNRVTLRDWFQLTLKEGLTVHRENLFSEEMTKTVTTRLSHVDLLRSVQ : 551
Pl_berghei : SIDFSFERILTVVGHEYFHNYTGNRVTLRDWFQLTLKEGLTVHRENLFSEETTKTATFRLTHIDLLRSVQ : 526
Pl_yoelii : SIDFSFERILTVVGHEYFHNYTGNRVTLRDWFQLTLKEGLTVHRENLFSEETTKTATFRLTHIDLLRSVQ : 526
Pl_chabaud : SIDFSFERILTVVGHEYFHNYTGNRVTLRDWFQLTLKEGLTVHRENLFSEQTTKTATFRLTHIDILRSVQ : 523
Pl_petteri : SIDFSFERILTVVGHEYFHNYTGNRVTLRDWFQLTLKEGLTVHRENLFSEQTTKTATFRLTHIDLLRSVQ : 523
Pl_vinckei : SIDFSFPRILTVVGHEYFHNYTGDRVTLRDWFQLTLKEGLTVHRENLFSEQTTKTATFRLTHIDLLRSVQ : 523
Pl_cynomol : SIDFSFERILTVVGHEYFHNYTGNRVTLRDWFQLTLKEGLTVHRENLFSEQTTKTATFRLDHVDILRSVQ : 549
Pl_vivax : SIDFSFERILTVVGHEYFHNYTGNRVTLRDWFQLTLKEGLTVHRENLFSEQTTKTATFRLDHVDILRSVQ : 551
Pl_knowles : SIDFSFERILTVVGHEYFHNYTGNRVTLRDWFQLTLKEGLTVHRENLFSEQTTKTATFRLDHVDLLRSVQ : 550
Pl_fragile : SIDFSFERILTVVGHEYFHNYTGNRVTLRDWFQLTLKEGLTVHRENLFSEQTTKTATFRLDHVDLLRSVQ : 548
Pl_reichen : SIDFSYARILTVVGHEYFHNYTGNRVTLRDWFQLTLKEGLTVHRENLFSEEMTKTVTTRLSHVDLLRSVQ : 553
Pl_gaboni : SIDFSYARILTVVGHEYFHNYTGNRVTLRDWFQLTLKEGLTVHRENLFSEEMTKTVTTRLSHVDLLRSVQ : 546
 **|**

**Arg489**

* 580 * 600 * 620 *
3EBG_A|PDB : FLEDSSPLSHPIRPESYVSMENFYTTTVYDKGSEVMRMYLTILGEEYYKKGFDIYIKKNDGNTATCEDFN : 426
Pl_falcipa : FLEDSSPLSHPIRPESYVSMENFYTTTVYDKGSEVMRMYLTILGEEYYKKGFDIYIKKNDGNTATCEDFN : 621
Pl_berghei : FLEDSSPLSHPIRPESYISMENFYTNTVYDKGSEVMRMYQTILGDDYYKKGIDIYLKKHDGGTATCEDFN : 596
Pl_yoelii : FLEDSSPLSHPIRPESYISMENFYTNTVYDKGSEVMRMYQTILGDEYYKKGIDIYLKKHDGGTATCEDFN : 596
Pl_chabaud : FLEDSSPLSHPIRPESYISMENFYTNTVYDKGSEVMRMYQTILGDEYYKKGISIYLKKHDGGTATCEDFN : 593
Pl_petteri : FLEDSSPLSHPIRPESYISMENFYTNTVYDKGSEVMRMYQTILGDEYYKKGISIYLKKHDGGTATCEDFN : 593
Pl_vinckei : FLEDSSPLSHPIRPESYISMDNFYTNTVYDKGSEVMRMYQTILGDEYYKKGITLYLKKHDGGTATCEDFN : 593
Pl_cynomol : FLEDSSPLAHPIRPESYVSMENFYTTTVYDKGSEVMRMYQTILGDEYYKKGMDIYIKKNDGGTATCEDFN : 619
Pl_vivax : FLEDSSPLAHPIRPESYVSMENFYTTTVYDKGSEVMRMYQTILGDEYYKKGMDIYIKKNDGGTATCEDFN : 621
Pl_knowles : FLEDSSPLAHPIRPESYVSMENFYTTTVYDKGSEVMRMYQTILGDDYYKKGMDIYIKKNDGGTATCEDFN : 620
Pl_fragile : FLEDSSPLAHPIRPESYVSMENFYTTTVYDKGSEVMRMYQTILGDEYYKKGMDIYIKKNDGGTATCEDFN : 618
Pl_reichen : FLEDSSPLSHPIRPESYVSMENFYTTTVYDKGSEVMRMYLTILGEEYYKKGFDIYIKKNDGNTATCEDFN : 623
Pl_gaboni : FLEDSSPLSHPIRPESYVSMENFYTTTVYDKGSEVMRMYLTILGEEYYKKGFDIYIKKNDGNTATCEDFN : 616
 **|**

**Glu572**

640 * 660 * 680 * 700
3EBG_A|PDB : YAMEQAYKMKKADNSANLNQYLLWFSQSGTPHVSFKYNYDAEKKQYSIHVNQYTKPDENQKEKKPLFIPI : 496
Pl_falcipa : YAMEQAYKMKKADNSANLNQYLLWFSQSGTPHVSFKYNYDAEKKQYSIHVNQYTKPDENQKEKKPLFIPI : 691
Pl_berghei : DAMNEAYQMKKGNTDENLDQYLLWFSQSGTPHVTAEYIYDENEKTFTINLSQITYPDDNQKEKYPLFIPV : 666
Pl_yoelii : DAMNEAYQMKNGNTDENLDQYLLWFSQSGTPHVTAEYIYDENEKTFTINLSQITYPDDNQKEKYPLFIPV : 666
Pl_chabaud : EAMNEAYQMKNGNKEENLDQYLLWFSQSGTPHVTAEYSYDANAKTFTIKLSQVTYPDDNQKEKFPLFIPV : 663
Pl_petteri : EAMNEAYQMKNGNNEDNLDQYLLWFSQSGTPHVTAEYSYDANAKTFTIKLSQVTYPDDNQKEKFPLFIPV : 663
Pl_vinckei : EAMNEAYQMKNGNNEKNLDQYLLWFSQSGTPHVTAEYIYDEKAKTFTINLSQITYPDDNQKEKHPLFIPV : 663
Pl_cynomol : DAMNEAYKLKKGDKTANLDQYLLWFSQSGTPHVTAEYSYDAGKKEFVIEVTQVTNPDPNQKEKKALFIPI : 689
Pl_vivax : DAMNEAYKMKKGDKTANLDQYLLWFSQSGTPHVTAEYSYDAGKKEFVIEVTQVTNPDPNQKEKKALFIPI : 691
Pl_knowles : DAMNEAYKLKKGDKTANLDQYLLWFAQSGTPHVTAEYSYDAGKKEFVIDITQVTHPDPNQKEKKALFIPI : 690
Pl_fragile : SAMNEAYKLKKGDRSANLDQYMLWFSQSGTPHVTAEYSYDAGKKEFVIEVTQVTQPDPNQKEKKALFIPI : 688
Pl_reichen : YAMEQAYKMKKADNSANLNQYLLWFSQSGTPHVSFKYNYDAEKKQYSIHVNQYTKPDENQKEKKPLFIPI : 693
Pl_gaboni : DAMEQAYKMKKADNSANLNQYLLWFSQSGTPHVSFKYNYDAEKKQYSIHVNQYTKPDENQKEKKPLFIPI : 686

* 720 * 740 * 760 *
3EBG_A|PDB : SVGLINPENGKEMISQTTLELTKESDTFVFNNIAVKPIPSLFRGFSAPVYIEDQLTDEERILLLKYDSDA : 566
Pl_falcipa : SVGLINPENGKEMISQTTLELTKESDTFVFNNIAVKPIPSLFRGFSAPVYIEDNLTDEERILLLKYDSDA : 761
Pl_berghei : KVGFISPKDGKDVIPETVLELKKDKESFVFQNVSEKPIPSLFREFSAPVYIKDNLTDEERIALLKYDSDA : 736
Pl_yoelii : KVGFISPKDGKDVIPEVVLELKKDKESFVFQNVSEKPIPSLFREFSAPVYIKDNLTDEERIALLKYDSDA : 736
Pl_chabaud : KVGLISPKDGKDVIPEVVLEFKKDKDTFVFENIEEKPIPSLFREFSAPVYIKDNLTDEERIILLKYDSDA : 733
Pl_petteri : KVGLISPKDGKDVIPEVVLEFKKDNDTFVFENIEEKPIPSLFREFSAPVYIKDNLTDDERIILLKYDSDA : 733
Pl_vinckei : KVGLINPKDGKDIISEVVLEFKKDKDTFVFENINEKPIPSLFREFSAPVYIKDNLTDEERITLLKYDSDA : 733
Pl_cynomol : RVGFINPHNGQDVIPEVTLEFKKDKEKFVFNNVNEKPIPSLFRGFSAPVYIKDNLTDAERILLLKYDTDA : 759
Pl_vivax : RVGFINPKNGQDVIPEVTLEFKKDKEKFIFNNVNEKPIPSLFRGFSAPVYIKDNLTDSERILLLKYDTDA : 761
Pl_knowles : RVGFINPHNGKEVIPEVTLEFKKDKEKFIFSNVNEKPIPSLFRGFSAPVYIKDNLTDSERIVLLKYDTDA : 760
Pl_fragile : RVGFINPHNGQDVIPEVTLEFKKEKEKFIFNNVNEKPIPSLFRGFSAPVYIKDNLTDAERILLLKYDSDA : 758
Pl_reichen : SVGLINPENGKEMISQTTLELTKESDTFVFNNIDVKPIPSLFRGFSAPVYIEDNLTDEERILLLKYDSDA : 763
Pl_gaboni : SVGLINPQNGKEMISQTTLELTKESDTFVFNNIDVKPIPSLFRGFSAPVYIEDNLTDEERILLLKYDTDA : 756

780 * 800 * 820 * 840
3EBG_A|PDB : FVRYNSCTNIYMKQILMNYNEFLKAK----NEKLESFQLTPVNAQFIDAIKYLLEDPHADAGFKSYIVSL : 632
Pl_falcipa : FVRYNSCTNIYMKQILMNYNEFLKAK----NEKLESFNLTPVNAQFIDAIKYLLEDPHADAGFKSYIVSL : 827
Pl_berghei : FVRYNVCIDLYMKQIIKNYNELISQKTK--ENNVLELSLTPVNDEFINAIKHLLEDKHADPGFKSYIIAL : 804
Pl_yoelii : FVRYNVCIDLYMKQIIKNYNELVSQKTK--ENDLVELSLTPVNDEFINAIKHLLEDKHADPGFKAYIIAL : 804
Pl_chabaud : FVRYNVCIDLYMKQIIKNYNEFLSQKTK--EANGLEHSLTPVSEDFINAIKHLLEDKHSDPGFKAYIIAL : 801
Pl_petteri : FVRYNVCIDLYMKQIIKNYNEFLAQKTK--ETDILEHSLTPVSEDFINAIKHLLEDKHADPGFKSYIIAL : 801
Pl_vinckei : FVRYNVCIDLYMKQIIKNYNEFLSQKTK--GTEILEHTLTPVSEDFINAIKHLLEDKHADPGFKSYIITL : 801
Pl_cynomol : FVRYNVCVDLYMKQILMNYQELLKAKTENKQESDEKPSLTPVSEDFINAIKYLMEDPHADAGFKSYIITL : 829
Pl_vivax : FVRYNVCVDLYMKQILKNYQELLQAKSENKQESAEKPSLTPVSEDFINAIKYLMEDPHADAGFKSYIITL : 831
Pl_knowles : FVRYNVCVDLYMKQIMKNYQELLQAKAENKQESTEKPKLTPVSEDFISAIKYLMEDPHADAGFKSYIITL : 830
Pl_fragile : FVRYNVCVDLYMKQIIKNYEELLQAKAQSKHEGAAKPKLTPVNEDFINAIEYLMKDPHADAGFKSYIVTL : 828
Pl_reichen : FVRYNSCTNIYMKQILMNYNEFLKAK----NEKLESFNLTPVNAQFIDAIKYLLEDPHADAGFKSYIVSL : 829
Pl_gaboni : FVRYNSCTNIYMKQILMNYNEFLKAK----NENLQSFNLTPVNTEFIDAIKYLLEDPHADAGFKSYIVSL : 822

**Lys849**

* 860 **|** * 880 * 900 *
3EBG_A|PDB : PQDRYIINFVSNLDTDVLADTKEYIYKQIGDKLNDVYYKMFKSLEAKADDLTYFNDESHVDFDQMNMRTL : 702
Pl_falcipa : PQDRYIINFVSNLDTDVLADTKEYIYKQIGDKLNDVYYKMFKSLEAKADDLTYFNDESHVDFDQMNMRTL : 897
Pl_berghei : PRDRYIMNYIKEVDPIVLADTKDYIYKQIGSRLNPVLFSIFQNTESKANDMTHFKDESYIDFDQLNMRKL : 874
Pl_yoelii : PRDRYIMNYIKEVDPIVLADTKDYIYKQIGSRLNPILFSIFQNTESKANDMTHFKDESYIDFDQLNMRKL : 874
Pl_chabaud : PRDRYIMNYIKEVDPIILADTKDYIYKQMGNRLNPILFSIFQDTESKANDMTHFKDESYVDFDQLNMRKL : 871
Pl_petteri : PRDRYIMNYIKEVDPIVLADTKDYIYKQMGNRLNPILFSIFQDTESKANDMSHFKDESYVDFDQLNMRKL : 871
Pl_vinckei : PRDRYIMNYIKEVDPIVLADTKDYIYKQIGNRLNPILFSIFQDSESKANDMTHFNDESFVDFDQLNIRSL : 871
Pl_cynomol : PRDRFIINYIKNVDTDVLADTKDFIYKQLGDKLNDLYFQIFKSIQAKADDMTHFEDESYVDFEQLNMRKL : 899
Pl_vivax : PRDRFILNYIKNVDTDVLADTKDFIYKQLGDKLNDLYFQMFKSLQAKADDMTHFEDESYVDFEQLNMRKL : 901
Pl_knowles : PRDRFIINSIRNVDTDVLADTKDFIYKQLGDKLNDLYFQIFKSIQAKADDMTHFEDESYVDFEQLNMRKL : 900
Pl_fragile : PKDRFIINYIKNVDTDVLADTKDFIYKQLGDKLNDLYFQIFKSIQAKADDMTHFEDESYVDFEQLNMRKL : 898
Pl_reichen : PQDRYIINFVSNLDTDVLADTKEYIYKQIGEKLNDVYYKMFKSLEAKADDLTYFNDESHVDFDQMNMRTL : 899
Pl_gaboni : PQDRYIINFVSNLDTDVLADTKEYIYKQIGDKLNDVYYKMFKSLEAKADDLTYFNDESHVDFDQMNMRTL : 892
 **|** **|**

**Asp830** **Glu850**
 920 * 940 * 960 * 980
3EBG_A|PDB : RNTLLSLLSKAQYPNILNEIIEHSKSPYPSNWLTSLSVSAYFD--KYFELYDKTYKLSKDDELLLQEWLK : 770
Pl_falcipa : RNTLLSLLSKAQYPNILNEIIEHSKSPYPSNWLTSLSVSAYFD--KYFELYDKTYKLSKDDELLLQEWLK : 965
Pl_berghei : RNSILMMLSKAQYPHMLKYIKEQSNSPYPSNWLASLSASSYFSGDDYYDLYDKTYKLSKNDELLLQEWLK : 944
Pl_yoelii : RNSILVMLSKAQYPHMLKYIKEQSKSAYPSNWLASLSASAYFSGDDYYDLYDKTYKLSKNDELLLQEWLK : 944
Pl_chabaud : RNSIMVMLSKAQYPHMLKYVKDQAQSPYPSNWLASLSASAYFTGDDYYNLYDKTYNLSKNDELLLQEWLK : 941
Pl_petteri : RNSIMVMLSKAQYPHMLKYIKEQAKSPYPSNWLTSLSASAYFSGDDYYDLYDKTYNLSKNDELLLQEWLK : 941
Pl_vinckei : RNSIMVMLSKAQYPHMLKYVKEQAKSPYPSNWLTSLTASAYFTGNDYYDLYDKTYNLSKNDELLLQEWLK : 941
Pl_cynomol : RNTLLTLLSRAKYPNMLDQIMEHSKSPYPSNWLASLAVSAYYD--KYFDLYEMTYNQSKDDELLLQEWLK : 967
Pl_vivax : RNTLLTLLSRAKYPNMLDQIMEHSKSPYPSNWLASLAVSAYYD--KYFDLYEKTYNQSKDDELLLQEWLK : 969
Pl_knowles : RNTLLTLLSKAKYPNMLDHIMEHSKSPYPSNWLASLAVSAYYD--KYFDLYEKTYNQSKDDELLLQEWLK : 968
Pl_fragile : RNTLLALLSRAKYPNMLEQIMEHSKSPYPSNWLASLAVSAYYD--EYFDLYQKTYNQSKDDELLLQEWLK : 966
Pl_reichen : RNTLLSLLSKAQYPNILNEIIEHSKSPYPSNWLTSLSVSAYFD--KYFELYDKTYKLSKDDELLLQEWLK : 967
Pl_gaboni : RNTLLSLLSKAQYPNILNEILEHSKSPYPSNWLTSLSVSAYFD--KYFELYDKTYQLSKDDELLLQEWLK : 960

**|**

**Lys907**

 * 1000 * 1020 * 1040 *
3EBG_A|PDB : TVSRSDRKDIYEILKKLENEVLKDSKNPNDIRAVYLPFTNNLRRFHDISGKGYKLIAEVITKTDKFNPMV : 840
Pl_falcipa : TVSRSDRKDIYEILKKLENEVLKDSKNPNDIRAVYLPFTNNLRRFHDISGKGYKLIAEVITKTDKFNPMV : 1035
Pl_berghei : TVSRSDRSDIYSIIKKLEVEILKDSKNPNNIRAVYLPFTSNLRAFNDISGKGYKLMANVIMKVDKFNPMV : 1014
Pl_yoelii : TVSRSDRSDIYSIIKKLEVEILKDSKNPNNIRAVYLPFTANLRAFNDISGKGYKLMADVIMKVDKFNPMV : 1014
Pl_chabaud : TVSRSDRSDIYNIIKKLETEILKDSKNPNNIRAVYLPFTSNLRAFNDISGKGYKLMADVIMKVDKFNPMV : 1011
Pl_petteri : TVSRSDRKDIYNIIKKLEVEILKDSKNPNNIRAVYLPFTSNLRAFNDISGKGYKLMADIIMKVDKFNPMV : 1011
Pl_vinckei : TVSRSDRKDIYNIIKKLEVEVLKDSKNPNIIRAVYLPFTANLRAFNDISGKGYKLMADIVMKVDKFNPMV : 1011
Pl_cynomol : TVSRSDRKDIYDIIKKLETEVLKDSKNPNEIRAVYLPFTYNLRYFNDISGKGYKMMADIIMKVDKFNPML : 1037
Pl_vivax : TVSRSDRKDIYDIIKKLETEVLKDSKNPNEIRAVYLPFTYNLRYFNDISGKGYKMMADIIMKVDKFNPMV : 1039
Pl_knowles : TVSRSDRKDIYDIIKKLENEVLKDSKNPNEIRAVYLPFTNNLRYFNDISGKGYKMMADIIMKVDKFNPMV : 1038
Pl_fragile : TVSRSDRKDIYDIIKKLENEVLKDSKNPNEIRAVYLPFTNNLRYFNDISGKGYKMMADIIMKVDKFNPMV : 1036
Pl_reichen : TVSRSDRKDIYEILKKLENEVLKDSKNPNDIRAVYLPFTNNLRRFHDISGKGYKLIAEVITKTDKFNPMV : 1037
Pl_gaboni : TVSRSDRKDIYEILKKLENEVLKDSKNPNDIRAVYLPFTNNLRRFHDISGKGYKLIAEVITKTDKFNPMV : 1030
 **|**

**Arg969**

1060 * 1080 * 1100
3EBG_A|PDB : ATQLCEPFKLWNKLDTKRQELMLNEMNTMLQEPQISNNLKEYLLRLTNK- : 889
Pl_falcipa : ATQLCEPFKLWNKLDTKRQELMLNEMNTMLQEPNISNNLKEYLLRLTNKL : 1085
Pl_berghei : ATQLCDPFKLWNKLDLKRQALMHDEMNRMLNMENISPNLKEYLLRLTNKM : 1064
Pl_yoelii : ATQLCDPFKLWNKLDLKRQALMHDEMNRMLSMENISPNLKEYLLRLTNKM : 1064
Pl_chabaud : ATQLCDPFKLWNKLDLKRQALMHDEMNRMLSMDNISPNLKEYLLRLTNKM : 1061
Pl_petteri : ATQLCDPFKLWNKLDLKRQELMHNEMNRMLSMDNISPNLKEYLLRLTNKM : 1061
Pl_vinckei : ATQLCDPFKLWNKLDLKRQALMHEEMNRMLSMDNISPNLKEYLLRLTNKM : 1061
Pl_cynomol : ATQLCDPFKLWNKLDQKRQDMMLNEMNRMLSMENISNNLK---------- : 1077
Pl_vivax : ATQLCDPFKLWNKLDQKRQDMMLNEMNRMLSMENISNNLKEYLLRLTNKL : 1089
Pl_knowles : ATQLCEPFKLWNKLDMKRQDMMLNEMNRMLSMENISNNLKEYLLRLTNKL : 1088
Pl_fragile : ATQLCEPFKLWNKLDLKRQDMMQKEMNRMLSMENISNNLKEYLLRLTNKL : 1086
Pl_reichen : ATQLCEPFKLWNKLDTKRQELMLNEMNTMLQESNISNNLKEYLLRLTNKL : 1087
Pl_gaboni : ATQLCEPFKLWNKLDTKRQELMLKEMNTMLQEPNISNNLKEYLLRLTNKL : 1080

**Figure 2S**. The alignment of M1 aminopeptidase sequences from malaria strains. Residues involved in the migration of the ligands from the C-terminal channel are highlighted in cyan. Glu572 and Arg325 forming a salt bridge are in pink.
