# Supplementary material for: Steered molecular dynamics simulations reveal critical residues for (un)binding of substrates, inhibitors and a product to the malarial M1 aminopeptidase
Source: PLoS Comput Biol. 2018 Oct 31;14(10):e1006525. doi: 10.1371/journal.pcbi.1006525 (PMC6239339; doi:10.1371/journal.pcbi.1006525)
Supplement: S1 Link — (DOCX) [file pcbi.1006525.s013.docx]

**S1 Link.** **Ligand-Residue Binding Network Script is located here:** <https://github.com/Danny221212/sMD-PF-M1AAP>
